# Genome-Wide Association Study of Kernel Traits in *Aegilops tauschii*

**DOI:** 10.3389/fgene.2021.651785

**Published:** 2021-05-28

**Authors:** Qing Wang, Ning Yan, Hao Chen, Sirui Li, Haiyan Hu, Yu Lin, Haoran Shi, Kunyu Zhou, Xiaojun Jiang, Shifan Yu, Caixia Li, Guangdeng Chen, Zisong Yang, Yaxi Liu

**Affiliations:** ^1^State Key Laboratory of Crop Gene Exploration and Utilization in Southwest China, Chengdu, China; ^2^Triticeae Research Institute, Sichuan Agricultural University, Chengdu, China; ^3^Chengdu Foreign Language School, Chengdu, China; ^4^School of Life Sciences and Technology, Henan Institute of Science and Technology, Xinxiang, China; ^5^College of Resources, Sichuan Agricultural University, Chengdu, China; ^6^College of Resources and Environment, Aba Teachers University, Wenchuan, China

**Keywords:** *Aegilops tauschii*, candidate gene, genetic diversity, GWAS, kernel traits, SNP

## Abstract

*Aegilops tauschii* is the diploid progenitor of the D subgenome of hexaploid wheat (*Triticum aest*ivum L.). Here, the phenotypic data of kernel length (KL), kernel width (KW), kernel volume (KV), kernel surface area (KSA), kernel width to length ratio (KWL), and hundred-kernel weight (HKW) for 223 *A. tauschii* accessions were gathered across three continuous years. Based on population structure analysis, 223 *A. tauschii* were divided into two subpopulations, namely T-group (mainly included *A. tauschii* ssp. *tauschii* accessions) and S-group (mainly included *A. tauschii* ssp. *strangulata*). Classifications based on cluster analysis were highly consistent with the population structure results. Meanwhile, the extent of linkage disequilibrium decay distance (*r*^2^ = 0.5) was about 110 kb and 290 kb for T-group and S-group, respectively. Furthermore, a genome-wide association analysis was performed on these kernel traits using 6,723 single nucleotide polymorphism (SNP) markers. Sixty-six significant markers, distributed on all seven chromosomes, were identified using a mixed linear model explaining 4.82–13.36% of the phenotypic variations. Among them, 15, 28, 22, 14, 21, and 13 SNPs were identified for KL, KW, KV, KSA, KWL, and HKW, respectively. Moreover, six candidate genes that may control kernel traits were identified (*AET2Gv20774800, AET4Gv20799000, AET5Gv20005900, AET5Gv20084100, AET7Gv20644900*, and *AET5Gv21111700*). The transfer of beneficial genes from *A. tauschii* to wheat using marker-assisted selection will broaden the wheat D subgenome improve the efficiency of breeding.

## Introduction

*Aegilops tauschii* (2n = 2× = 14, DD) is the diploid progenitor of the D subgenome of hexaploid wheat (*Triticum aestivum* L., 2n = 6× = 42, AABBDD) and a vital genetic resource for the improvement of wheat quality and yield (Dvořák et al., 1998; Ogbonnaya et al., 2013). The *A. tauschii* has a rich genetic diversity and multiple biological and abiotic resistances, including excellent genetic resources such as stress resistance (Qin et al., 2016), disease resistance (Zhang et al., 2019), and improved yield, which are uncommon in ordinary hexaploid wheat.

Hexaploid wheat arose through natural hybridization and chromosome doubling between a cultivated allotetraploid (2n = 4× = 28, AABB) and *A. tauschii* (Dvořák et al., 1998; Matsuoka, 2011). However, common wheat descends from a small number of spontaneous interspecific hybrids (Cox, 1997). Thus, there is scope for *A. tauschii* to improve wheat and increase wheat yield by artificially synthesizing hexaploids. After several collaborative long-time research efforts, the International Center for Maize and Wheat Improvement (CIMMYT) have synthesized hexaploid wheat lines by crossing elite tetraploid durum with *A. tauschii* (Ogbonnaya et al., 2013). The *A. tauschii* has several yield traits or components that may be transferred to synthetic hexaploid wheat when used as a paternal parent. Furthermore, it has previously been used to introgress yield traits into wheat, such as the large-kernel wheat Chuanmai 42 (Zhang et al., 2004) and heavy panicle Shumai 830 (Hao et al., 2019). With the rapid development of scientific research technology, identification of the linked markers in the genetic background of *A. tauschii* can enable targeted introgressions, thus making it economical.

Single nucleotide polymorphism (SNP) is a third-generation genetic marker technology. SNPs are abundant and have traits such as high frequency and good genetic stability. Currently, SNP genetic studies are widely used for kernel yield, disease resistance, and stress resistance. Genome-wide association study (GWAS) based on linkage disequilibrium (LD) has been widely adopted to identify loci significantly associated with important and complex morphological traits in several species, including *A. tauschii* (Liu et al., 2015a,b), rice (Chen et al., 2014), wheat (Lin Y. et al., 2017, 2019, 2020a; Liu Y. et al., 2017), and maize (Lu et al., 2010, 2011 Yang et al., 2014). Moreover, only a few GWAS have reported kernel size traits in *A. tauschii*. For example, using 193 *A. tauschii* accessions worldwide, 58 SSR were identified in three environments for seven grain traits (Zhao et al., 2015). Using 5,249 SNPs, a GWAS was performed for 114 *A. tauschii* germplasm, and a total of 17 SNPs associated with grain size traits distributed over all the seven chromosomes (Arora et al., 2017). However, this study aimed to investigate marker-trait associations for kernel size traits using SNPs in a core collection of 223 *A. tauschii* of diverse origin. Moreover, our objective was to scan candidate gene responses to kernel size traits. These identified genes and SNPs will provide an important research framework for cloning kernel trait genes in *A. tauschii*.

## Materials and Methods

### Plant Materials and Field Experiments

A total of 223 *A. tauschii* accessions were collected by Triticeae Research Institute, Sichuan Agricultural University. These *A. tauschii* accessions were originally obtained from 17 different countries (Supplementary Table 1). According to morphological classification criteria (Zhao et al., 2018), 135 and 88 *A. tauschii* accessions were classified as *A. tauschii* ssp. *tauschii* and *A. tauschii* ssp. *strangulata*, respectively (Supplementary Table 1).

All *A. tauschii* were planted in Wenjiang, Chongzhou, and Wenjiang in 2017, 2018, and 2019, respectively. Each accession was planted in three rows. Each row’s length was 1.5 m, and the space between the rows was 0.6 m, as a previous study described (Liu et al., 2015b). Spikes were harvested at physiological maturity and threshed by hand. Fifty kernels of each *A. tauschii* plant were used to evaluate six traits with three repetitions. Kernel length (KL), kernel width (KW), kernel width to length ratio (KWL), kernel surface area (KSA), and kernel volume (KV) were evaluated in all three environments, and hundred-kernel weight (HKW) was evaluated in 2018 and 2019. Kernel morphologic traits, including KL, KW, KWL, KSA, and KV, were scanned using an Epson XL scanner system (11,000 ×) (Seiko Epson Corporation, Nagano-ken, Japan) and analyzed using the Win-SEEDLE Pro 2012a image analysis system (Régent Instruments, Quebec, Canada) software. Hundred-kernel weight was calculated as two times the weight of 50 kernels.

### Statistical Analysis of Phenotypic Data

Analysis of variance (ANOVA) was conducted using the “car” package in the software R 3.5.1 R Core Team (2014). As HKW was only calculated in two environments, ANOVA could not be conducted for HKW. In this study, we established selection indices involving multiple kernel traits, and a series of linear regressions were performed for all traits. We built a series of linear regressions to explain HKW and chose our predictive variables through a stepwise selection process.

The broad-sense heritability was calculated using the Smith et al. (1998) method as previous studies described (Liu Y. et al., 2017; Lin et al., 2020b; Lin Y. et al., 2021). Meanwhile, to reduce the environmental impact on kernel traits, best linear unbiased predictors (BLUP) of each trait across environments were calculated using SAS 9.2 (SAS Institute Inc., Cary, NC). Descriptive analysis, Pearson’s correlation, linear regression, and clustering analyses were performed based on BLUP values for each trait using SPSS 20 (IBM, United States). Moreover, three different categories were calculated based on traits, i.e., low-, mid-, and high-performing genotypes corresponding to below, between, and above X ± SD (Standard Deviation), respectively (Zar, 2010; Abdel-Ghani et al., 2012), where X represent mean values of each trait. Meanwhile, Shannon–Weaver diversity index (*H’*) was calculated based on BLUP values for six kernel traits using the formula.

$$H^{\prime }=-\sum_{i=1}^{n}P_{i}Ln\left(Pi\right)$$

Where *P*_*i*_ is the number of materials in the *i* level of a specific trait in the total percentage of copies, and Ln is the natural logarithm (Hutcheson, 1970).

### Genotyping and Genetic Diversity Analysis

Genomic DNA from each *A. tauschii s*amples was extracted from the young leaves using the CTAB method (Murray and Thompson, 1980). All *A. tauschii* samples were genotyped by Illumina 10K SNP array, and the gathered SNPs were mapped onto the *A. tauschii* reference genome v4.0 (Aet v4.0^1^) to obtain the physical location (Luo et al., 2017). Then, the mapped SNPs with minor allele frequency (MAF) >5% and missing data <20% were retained for further analysis. Finally, a total of 6,723 polymorphic markers were obtained and used for population structure, kinship, and association analysis. Genetic diversity was evaluated using polymorphism information content (PIC), as PIC = 1−Σ(Pi)^2^, where Pi is the proportion of the population carrying the allele (Botstein et al., 1980).

### Population Structure, Kinship and Linkage Disequilibrium Analysis

Population structure was analyzed using the Bayesian inference program STRUCTURE 2.3.4 based on the linkage ancestry model (Pritchard et al., 2000; Falush et al., 2007). A total of 10,000 burn-in iterations followed by 10,000 Markov Chain Monte Carlo iterations for K = 1–10 clusters were used to identify the optimal range of K, performing 10 runs per K. The optimal value of K was determined using STRUCTURE HARVESTER (Earl and vonHoldt, 2012) based on the Evanno method (Evanno et al., 2005). The CLUMPP (Jakobsson and Rosenberg, 2007) was used to determine the best comparison among five repeated samples. Kinship was estimated using 6,723 markers in TASSEL 3.0 (Bradbury et al., 2007). The LD squared allele frequency correlation (*r*^2^), which contains both mutational and recombination history, as evaluated for linked/syntenic loci (*p* < 0.001). The LD analyses was conducted separately for the T-group and S-group, respectively. The LD estimates between marker pairs were obtained using TASSEL 3.0, the mean *r*^2^ over different genetic distances was calculated for the T-group and S-group, respectively.

### Genome-Wide Association Analysis and Candidate Gene Prediction

Genome-wide association analysis was performed based on 6,723 SNPs using mean value of each environment and the BLUP values of each trait in Tassel 3.0 based on a mixed linear model (MLM) (Bradbury et al., 2007). The significance threshold was set at p -value < 0.001, correspondingly −log_1__0_^(^*^*p*^*^)^ = 3.00 as previous studies (Liu J. et al., 2017; Ye et al., 2019; Fu et al., 2020). Manhattan and Quantile-Quantile plots of GWAS results were plotted in R 3.5.1 (R Core Team., 2014).

Based on Aet v4.0, putative genes in 10 Kb upstream and downstream of the significant SNPs were selected and then annotated using KEGG Orthology Based Annotation System 3.0 (KOBAS 3.0) (Xie et al., 2011; Arora et al., 2017; Wu et al., 2017). Arabidopsis and rice were used as background species. Candidate genes were identified according to the homologous function.

## Results

### Marker Distribution and Population Structure Analysis

A total of 6,723 polymorphic SNPs was mapped on the *A. tauschii* reference genome Aet v4.0 with MAF >5%, missing data <20%. The 6,723 SNPs were evenly distributed on seven chromosomes of *A. tauschi* (Supplementary Figure 1). The number of SNPs ranged from 784 for chromosome 4D to 1,231 for chromosome 2D (Supplementary Table 2). The marker density ranged from 0.53 to 0.70 Mb for each chromosome (chromosomes 2 and 6D, respectively) (Supplementary Table 2). The PIC ranged from 0.10 to 0.50, with an average value of 0.42 for the whole subgenome (Supplementary Table 2), indicating a high polymorphism of SNPs.

Based on the population structure analysis, *K* = 2 was selected. Thus, the whole panel was divided into two groups (Supplementary Table 1). Group 1 (S-group) contained 84 *A. tauschii*, including 83 *A. tauschii* ssp. *strangulata* and one *A. tauschii* ssp. *tauschii*. Group 2 (T-group) contained 139 *A. tauschii*, including 137 of *A. tauschii* ssp. *tauschii* and two *A. tauschii* ssp. *strangulata*. Meanwhile, the LD analyses were conducted separately for the T-group and S-group two lineages. The mean *r*^2^ values gradually decreased with increasing pairwise distance. The extent of LD decay distance (*r*^2^ = 0.5) was about 110 and 290 kb for T-group and S-group, respectively (Supplementary Figure 2).

### Phenotypic Variation and Cluster Analysis

**FIGURE 1:**
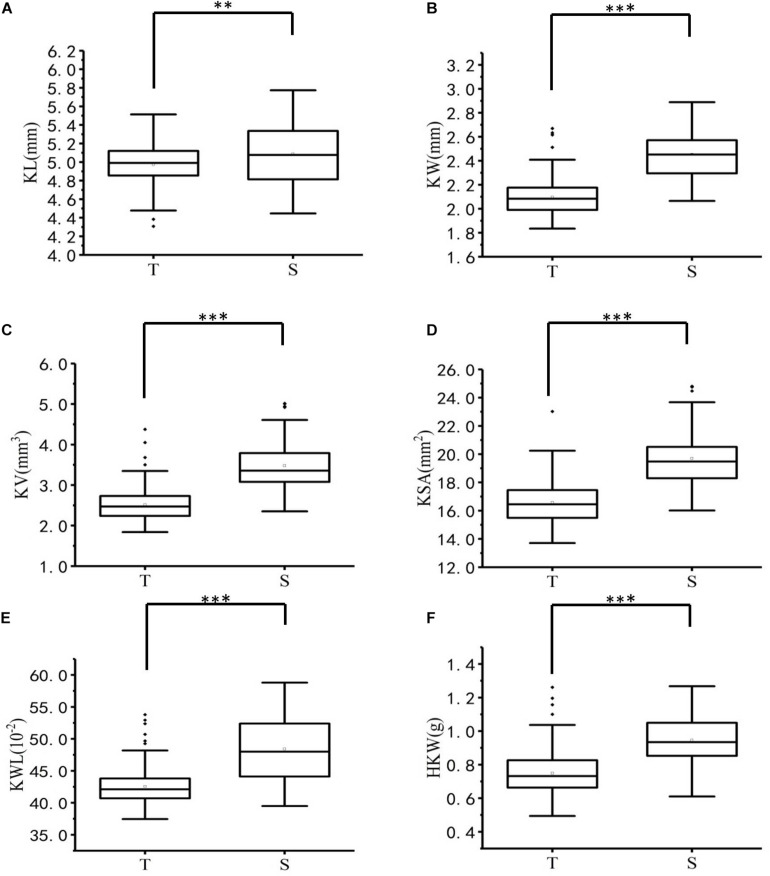
Boxplots of six kernel characters of T-group and S-group. ^∗∗^, and ^∗∗∗^ denote significance at *p* < 0.01 and *p* < 0.001, respectively. **(A)** KL, kernel length (mm), **(B)** KW, kernel width (mm), **(C)** KV, kernel volume (mm^3^), **(D)** KSA, kernel surface area (mm^2^), **(E)** KWL, kernel width to length ratio (%), **(F)** HKW, hundred-kernel weight (g).

The ANOVA results for 223 *A. tauschii* samples are listed in Table 1. All kernel traits showed significant (*p* < 0.001) differences among genotypes and environments, except for HKW. The coefficients of variation of the six kernel traits among three environments ranged from 8.49 to 48.99% (Supplementary Table 3). The heritability ranged from 0.74 for KL to 0.87 for KW, indicating medium to high heritability (Table 2). Based on BLUP values, coefficient of variation of six kernel traits ranged from 5.13 to 23.49% (Table 2). The minimum, maximum, and average values of KL, KW, KV, KSA, KWL, and HKW in the S-group were significantly (*p* < 0.01) higher than those in the T-group (Supplementary Table 4), and there were significant differences between the two subspecies (Figure 1). Results indicated that the six kernel traits in S-group exhibited higher *H’* values than those in T-group, and S-group subspecies had a wider diversity range than those in T-group subspecies. Regarding the phenotypic distribution of six kernel traits based on BLUP values, all traits frequency distribution was continuous (Supplementary Figure 3), indicating that kernel traits were quantitative and controlled by multiple genes.

**TABLE 1:** The analysis of variance of six kernel traits of 223 *Aegilops tauschii* among three environments.

	Type III sum of square		Mean square		*F*-values		Significance^†^	
	Environment	Genotype	Environment	Genotype	Environment	Genotype	Environment	Genotype
DF	2	222	2	222	2	222	2	222
KL	29.18	82.05	14.59	0.37	150.61	3.82	***	***
KW	25.33	50.49	12.67	0.23	442.70	7.95	***	***
KV	662.19	475.14	331.10	2.14	798.22	5.16	***	***
KSA	10556.28	5703.71	5278.14	25.69	933.59	4.54	***	***
KWL	1.49	2.13	0.74	0.01	542.37	7.00	***	***
HKW	−	−	−	−	−	−	−	−

DF, degrees of freedom; KL, kernel length; KW, kernel width; KV, kernel volume; KSA, kernel surface area; KWL, kernel width to length ratio; HKW, hundred-kernel weight. ^†^ ***, significant at p < 0.001, respectively. KL, KW, KV, KSA, and KWL were measured for 3 years, while HKW was measured for 2 years.

**TABLE 2:** Descriptive analysis, coefficient of variation, heritability, and Shannon–Weaver diversity index (*H*′) of six kernel traits based on BLUP values among the 223 *Aegilops tauschii*.

Trait	Mean ± SD	CV%	Min	Max	Heritability	*H*′		
						223 accessions	T-group^†^	S-group
KL (mm)	5.02 ± 0.26	5.13	4.31	5.77	0.74	0.85	0.76	0.98
KW (mm)	2.23 ± 0.24	10.70	1.83	2.89	0.87	0.89	0.79	0.93
KV (mm^3^)	2.88 ± 0.68	23.49	1.84	5.01	0.81	0.80	0.76	0.77
KSA (mm^2^)	17.75 ± 2.27	12.77	13.71	24.81	0.78	0.81	0.83	0.85
KWL (/)	0.45 ± 0.05	10.71	0.37	0.59	0.86	0.81	0.79	0.90
HKW (g)	0.82 ± 0.16	20.05	0.49	1.27	0.80	0.86	0.72	0.85

**CV, coefficient of variation; KL, kernel length; KW, kernel width; KV, kernel volume; KSA, kernel surface area; KWL, kernel width to length ratio; HKW, hundred-kernel weight; SD, standard deviation. ^†^ T-group and S-group were divided based on population structure analysis.**

Based on the BLUP value, correlation analysis for each trait showed significant correlations among traits, with correlation coefficients ranging from 0.27 (between KL and KW) to 0.98 (between KSA and KV) (Table 3). All correlations were positive, except for that between KL and KWL. KW showed medium-to-high correlations with KV, KSA, KWL, and HKW, while KL showed low-to-medium correlations with KV, KSA, KWL, and HKW (Table 3). Additionally, KW and KL showed a medium-to-high correlation with HKW. These results indicated that KW and KL were the major determinants of kernel architecture and weight in *A. tauschii*, showing that the same loci may orchestrate the control of these traits, indicating that SNPs identified in our study may play pleiotropic effects. The final model based on HKW phenotypic variation explained 76.8% of variability with KV, KL, KSA, and KW, verifying the above results (Supplementary Table 5). Meanwhile, cluster analysis (Ward’s method) grouped the 223 *A. tauschii* into two clusters (Supplementary Table 1). Results showed a high consistency of classification results by population structure.

**TABLE 3:** The correlation analysis of the six kernel traits based on best linear unbiased prediction (BLUP) values.

Trait	KL	KW	KV	KSA	KWL	HKW
KL	1					
KW	0.27**	1				
KV	0.47**	0.96**	1			
KSA	0.61**	0.90**	0.98**	1		
KWL	−0.30**	0.83**	0.67**	0.53**	1	
HKW	0.56**	0.79**	0.84**	0.84**	0.46**	1

KL, kernel length; KW, kernel width; KV, kernel volume; KSA, kernel surface area; KWL, kernel width to length ratio; HKW, hundred-kernel weight. ** represented significance at p < 0.01 respectively.

### Marker Trait Associations for Kernel Size Traits

**FIGURE 2:**
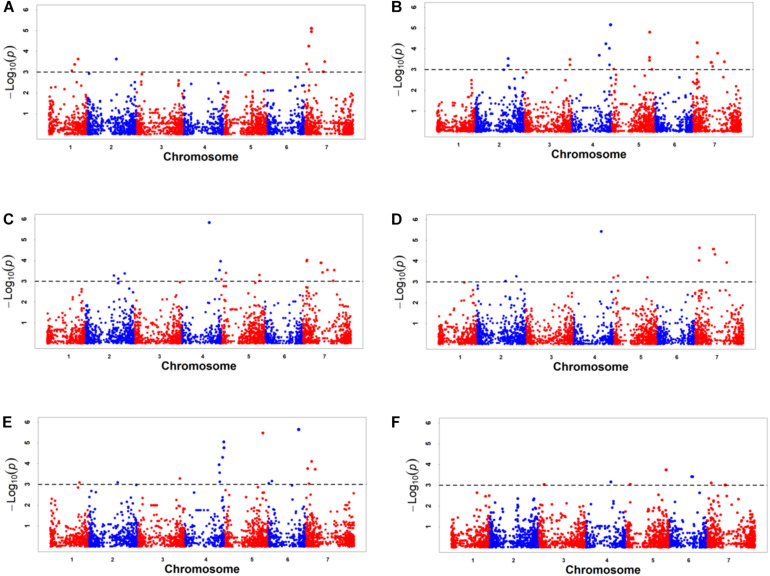
Manhattan plots of genome-wide association study results for six kernel traits based on BLUP value. **(A)** KL, kernel length, **(B)** KW, kernel width, **(C)** KV, kernel volume, **(D)** KSA, kernel surface area, **(E)** KWL, kernel width to length ratio, **(F)** HKW, hundred-kernel weight.

**FIGURE 3:**
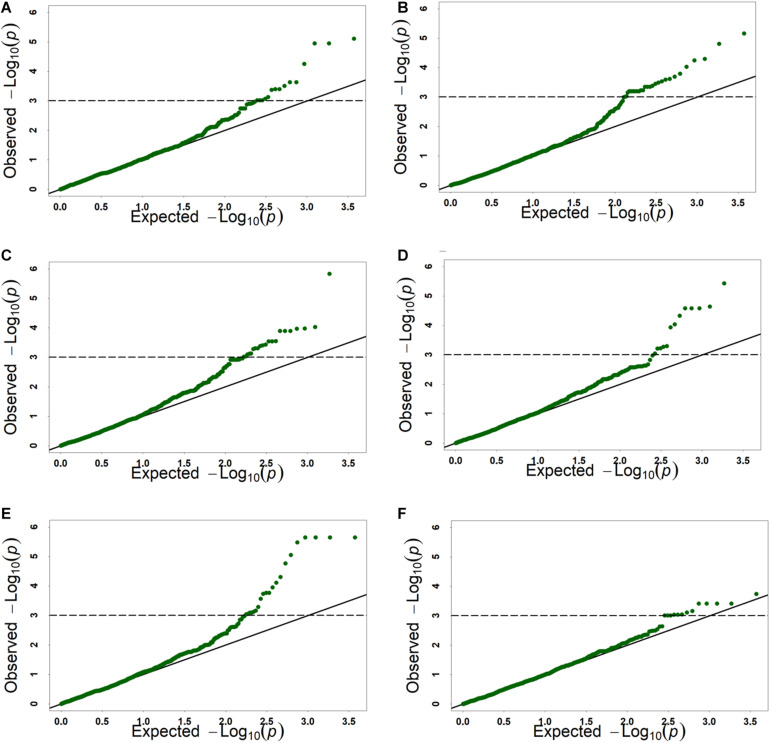
The Q-Q plots of genome-wide association study results for six kernel traits based on BLUP value. **(A)** KL, kernel length, **(B)** KW, kernel width, **(C)** KV, kernel volume, **(D)** KSA, kernel surface area, **(E)** KWL, kernel width to length ratio, **(F)** HKW, hundred-kernel weight.

GWAS was performed on all six traits using 6,723 SNPs among three environments. A total of 141 significant SNPs were identified for six kernel traits with phenotypic variation explained (PVE) ranging from 4.82 to 17.14% (Supplementary Table 6). The highest number of markers was detected for KV(78), which was followed by kernel volume KW (43), KSA (42), KWL (34), KL (26), HKW (21) (Supplementary Table 6). Based on BLUP values, GWAS was performed on all six traits using 6,723 SNPs by MLM. A total of 66 significant SNPs was identified for six kernel traits with phenotypic variation explained (PVE) ranging from 4.82 to 13.36% (Table 4 and Supplementary Table 6), and these markers were distributed on all seven chromosomes (Figures 2, 3, Table 4, and Supplementary Table 6). In order to reduce environment effects, significant SNPs detected using BLUP values were used for further analysis.

**TABLE 4:** Significant SNP markers identified for six kernel-related traits by genome-wide association study based on best linear unbiased prediction (BLUP) values.

Trait	Number	Chromosome	Mean −log_1__0_*^(*p)*^*	−log_1__0_*^(*p)*^* range	Mean PVE (%)	PVE range (%)
KL	15	1D/2D/7D	3.70	3.02–5.11	6.14	4.85–9.02
KW	28	2D/3D/4D/5D/7D	3.54	3.01–5.16	5.84	4.83–8.73
KV	22	2D/4D/5D/7D	3.74	3.02–6.64	6.22	4.82–13.02
KSA	14	2D//4D/5D/7D	4.22	3.05–6.83	7.13	4.87–13.36
KWL	21	1D/2D/3D/4D/5D/6D/7D	4.14	3.03–5.65	6.92	4.86–9.66
HKW	13	3D/4D/5D/6D/7D	3.22	3.01–3.74	5.27	4.82–7.05

**KL, kernel length; KW, kernel width; KV, kernel volume; KSA, kernel surface area; KWL, kernel width to length ratio; HKW, hundred-kernel weight; PVE, phenotypic variation explained.**

Based on BLUP values, 15 significant SNPs for KL were detected with PVE, ranging from 4.85 to 9.02%, these SNPs were distributed on chromosomes 1D, 2D, and 7D (Table 4 and Supplementary Table 6). For KW, 28 significant SNPs were detected with PVE, ranging from 4.83 to 8.73%. These SNPs were distributed on chromosomes 2D, 3D, 4D, 5D, and 7D. For KV, 22 significant SNPs were detected with PVE, ranging from 4.82 to 13.02%. These SNPs were distributed on chromosomes 2D, 4D, 5D, and 7D. For KSA, 14 significant SNPs were detected with PVE, ranging from 4.87 to 13.36%. These SNPs were distributed on chromosomes 2D, 4D, 5D, and 7D. For KWL, 21 significant SNPs were detected with PVE, ranging from 4.86 to 9.66%. A total of 13 significant SNPs were detected for HKW with PVE ranging from 4.82 to 7.05%. These SNPs were distributed on chromosomes 3D, 4D, 5D, 6D, and 7D. These SNPs were distributed on all seven chromosomes (Table 4 and Supplementary Table 6). The *contig17143_54*, located on chromosome 5D at 538.15 Mb, was strongly associated with KV with 13.02% PVE (Supplementary Table 6). The c*ontig 17143_54*, located on chromosome 5D at 538.15 Mb, was most significant for KSA with 13.36% PVE, while *contig67633_66*, located on chromosome 6D at 406.04 Mb, was most significant with KL with 9.66% PVE. Twenty-six loci of six kernel traits showed pleiotropy, e.g., *contig17143_54*, located on chromosome 5D at 538.15 Mb, was significantly related to KW, KSA, KV, and HKW; *GDRF1KQ01CJ4KM_378*, located on chromosome 7D at 246.23 Mb, was significantly related to HKW, KSA, KL, KV, KW, and *F1BEJMU01CNNGZ_79*, located on chromosome 4D at 453.78 Mb, was significantly related to KW, KWL, KV.

### Candidate Genes That May Be Linked to Kernel Traits

Based on Aet v4.0, putative genes in 10 Kb upstream and downstream of the significant SNPs were homologous comparison using KOBAS 3.0. A total of 38 predicted genes were selected. Thirty-six and 38 genes were homologous to arabidopsis and rice, respectively (Supplementary Table 7). Six predicted genes, included *AET2Gv20774800*, *AET4Gv20799000*, *AET5Gv20005900*, *AET5Gv20084100*, *AET7Gv20644900*, and *AET5Gv21111700*, were homologous to *MST1* (Takeda et al., 2001; Mao et al., 2011), *MAC3B* (Monaghan et al., 2009; Li S. et al., 2018), *ETR1* (Yin et al., 2017), *ZAR1* (Guo et al., 2013; Yu et al., 2016), *NAC047* (Kunieda et al., 2008; Mathew et al., 2016), *EXPA7* (Lizana et al., 2010; Jadamba et al., 2020), respectively (Table 5). These genes (*MST1*, *MAC3B*, *ETR1*, *ZAR1*, *NAC047*, *EXPA7*) could affect embryo development, cause seed surface atrophy, increase the number of cells to increase organ size, or affect kernel size through ethylene response. Thus, the six *A. tauschii* genes maybe directly or indirectly regulate kernel growth or regulate kernel size.

**TABLE 5:** Candidate genes identified for six kernel traits.

A. tauschii gene	Marker	Chr	Position (Mb)	Trait	Rice gene	Arabidopsis gene	Putative candidate genes
*AET2Gv20774800*	*GBF1XID01 D2CAC_283*	2D	438.95	KW		*MST1*	Monosaccharide transporters (*MST1*)
*AET4Gv20799000*	*GA8KES401 CWBR7_178*	4D	501.57	KW, KWL		*MAC3B*	U-Box Proteins (*MAC3B*)
*AET5Gv20005900*	*be405667Contig 1ATwsnp1*	5D	2.89	KV, KSA		*ETR1*	Ethylene receptor protein (*ETR1*)
*AET5Gv20084100*	*F5XZDLF01A U4HH_125*	5D	32.51	KW		*ZAR1*	RLK/Pelle kinase family (*ZAR1*)
*AET7Gv20644900*	*GDRF1KQ01C J4KM_378*	7D	246.23	KW, KL, HKW, KV, KSA		*NAC047*	*NAC* Family Proteins
*AET5Gv21111700*	*contig1 7143_54*	5D	538.15	KW, KV, KSA, HKW	*EXPA7*		Expansin genes (*EXPA7*)

**Chr, chromosome; KL, kernel length; KW, kernel width; KV, kernel volume; KSA, kernel surface area; KWL, kernel width to length ratio; HKW, hundred-kernel weight.**

## Discussion

The improvement of common wheat has gone through the cross between landraces and main popularized varieties to the cross between elite varieties now. However, wheat has been affected by domestication and selection of long-term backbone parents, and genetic “evolutionary bottlenecks” have appeared, which leads to a decrease in yield. Wheat kernel traits are the most important factor affecting yield, and excellent kernel traits greatly increase yield. However, *A. tauschii* is one of the ancestral species of hexaploid wheat and the donor species of D subgenome. It has a lot of valuable genes and a rich genetic diversity, and a vital genetic resource for the improvement of wheat quality and yield (Dvořák et al., 1998; Ogbonnaya et al., 2013). The purpose of this research is to dig out the excellent genes that regulate kernel size in *A. tauschii*, and lay a foundation for the transfer of common wheat and the broadening of genetic diversity. There have been many successful cases of introducing *A. tauschii* genes into wheat through hybridization. Such as Chuanmai 42, Shumai 969, and Shumai 830 etc. thereinto, Chuanmai 42 is a large and heavy spike cultivar with a large kernel (Zhang et al., 2004; Duan et al., 2006), and Shumai 830, is also a heavy spike cultivar (Hao et al., 2019). These successful wheat varieties suggest the considerable potential of *A. tauschii* for wheat improvement, especially for breeding cultivars with large and heavy spikes. However, the aim of this study was to discover genes in *A. tauschii* that regulate kernel size, introducing the significant target markers that affect kernel traits directly into wheat would accelerate the breeding of target varieties and save time.

In this study, 223 *A. tauschii* were divided into T-group and S-group subgroups through population STRUCTURE. T-group and S-group representing *A. tauschii* ssp. *tauschii* and *A. tauschii* ssp. *strangulata*, respectively. The results of this study are completely consistent with previous studies (Arora et al., 2017). Population structure is one of the most important factors affecting LD (Flint-Garcia et al., 2003). Thus, the LD analyses was conducted separately for the T-group and S-group, respectively. Only one previous study reported LD decay distance in *A. tauschii*. The LD decay distance was reported at 9.8 and 2.7 cM for T-group and S-group, respectively (Arora et al., 2017). This study firstly reported the LD decay distance based on physical distance. The LD decay distance was highest in the T-group (approximately 110 kb) and S-group (approximately 290 kb), the average LD decay distance was approximately 200 kb. In wheat, the LD decay distance was 250 kb for D subgenome, consistent with our results (Long et al., 2019).

The present study aimed to identify significant markers for kernel size trait in *A. tauschii*, the D subgenome donor of hexaploid wheat. Significant (*p* < 0.001) differences were noted among genotypes and environments; *A. tauschii* showed high diversity, indicating high research and utilization value, and it could make a major contribution to broadening the genetic diversity of the wheat D subgenome. In the present study, heritabilities of kernel traits were medium to high. In previous studies, moderate or high heritabilities were also observed in *A. tauschii* (Arora et al., 2017), consistent with our study. In some reports in wheat, moderate to high heritability were also observed (Kuchel et al., 2007; Liu Y. et al., 2017). These results indicated that the kernel size-related traits was more controlled by genetic factors. We used correlation and linear regression analyses revealed a significant positive correlation between KL, KW, KV, and HKW, and HKW increased with increasing KL, KW, and KV. Previous studies have found that, in the tetraploid and hexaploid wheat, *TaGW2-A1* mutations could increase KW and KL, thereby increasing yield by increasing TKW (Simmonds et al., 2016). The correlations between these trait points to a causal relationship between kernel size and weight because longer and wider kernels can accumulate more starch and, therefore, have greater kernel weight (Duan and Sun, 2005). Our study showed the consistent results. Thus, in the process of breeding, breeders could pay attention to discovering varieties with long or wide kernels. The identified *A. tauschii* accessions with long or wide kernels could be used in further breeding through SHW to broaden the genetic diversity of wheat.

In the present study, cluster analysis results were highly consistent (83%) with the population structure results. Similar results have been reported in *A. tauschii* and wheat (Liu et al., 2015b; Liu Y. et al., 2017). A previous cluster analysis performed using 29 morphological traits in 322 *A. tauschii* showed 72% consistency with the population structure results (Liu et al., 2015b). This result may be caused by an intermediate type between *A. tauschii* ssp. *tauschii* and *A. tauschii* ssp. *strangulata* subspecies (Zhao et al., 2018). Indeed, Kihara et al. (1965) reported that intermediate forms and hybrids existed between the two subspecies. Due to the presence of intermediate type or hybrids, there may be uncertainty in morphological identification, resulting in differences between morphology- and genotype-based identification. Besides, morphological traits may also be more easily affected by the ecological environment. Plant growth will be affected to varying degrees in different ecological environments, affecting plant growth and, ultimately, kernel size (Wang et al., 2009; Liu et al., 2015b). Thus, the morphological traits clustering is roughly correct, but there will be some classification errors. Compared with genotype data classification results, cluster analysis could correctly classify most *A. tauschii*.

For GWAS results, the threshold is set to Bonferroni correction method a = 0.01 or 0.05. Because MLM is too strict and can lead to over-correction. The Bonferroni correction method a = 1 to reduce negative errors caused by overcorrection (Yang et al., 2014). The *p*-value was 1.49 × 10^–4^ for the 6,723 SNPs, with a corresponding −log_1__0_*^(*p*)^* = 3.80. However, only 22 significant SNPs were identified. This is due to overcorrection caused by MLM. In addition, −log_1__0_*^(*p)*^* = 3.00 is also commonly set as a threshold (Liu J. et al., 2017; Ye et al., 2019; Fu et al., 2020) to reduce the negative false rate. Meanwhile, it has also been successfully applied in GWAS in wheat (Liu J. et al., 2017; Ye et al., 2019; Fu et al., 2020). This indicates that this threshold is a frequently used empirical value and the results are reliable. In this study, the threshold setting as −log_1__0_^(^*^*p*^*^)^ = 3.00, 66 significant loci were identified for kernel traits using 6,723 SNPs markers by GWAS. To the best of our knowledge, to date, only two studies have reported QTL for kernel characteristic traits in *A. tauschii* based on GWAS (Zhao et al., 2015; Arora et al., 2017). Owing to the lack of publicly available marker sequence information, our results were compared to those of previous studies based on chromosomes. In previous studies for KL, significant loci were identified on chromosomes 1D, 2D, 5D, and 6D (Zhao et al., 2015; Arora et al., 2017), while in this study, 11 significant markers were identified on chromosome 7D; these may represent novel loci. For KW, we detected 28 significant markers on chromosomes 2D, 3D, 4D, 5D, and 7D. Significant markers on these chromosomes were also found in previous studies (Zhao et al., 2015; Arora et al., 2017). For KSA, 14 significant markers were identified on chromosomes 2D, 4D, 5D, and 7D, and loci on chromosome 4D may be novel according to a comparison with previous research (Zhao et al., 2015). For HKW, loci were identified on all chromosomes in previous studies, except for 7D (Zhao et al., 2015; Arora et al., 2017). In this study, significant markers were identified on chromosome 7D, as well as on chromosomes 3D, 4D, 5D, and 6D. Besides, 21 significant markers for KWL were identified on all seven chromosomes, and 22 for KV were identified on chromosomes 2D, 4D, 5D, and 7D. GWAS is an important and effective approach for wheat breeding by helping in the design of hybrid crosses. The identified significant markers/variants can be designed for use as molecular markers in wheat breeding directly.

To further serve the breeding of target varieties with desirable kernel traits, six candidate genes were identified based on homologous functions in arabidopsis and rice. *AET2Gv20774800* was flanking the SNP marker *GBF1XID01D2CAC_283*. This gene was homologous to the *MST1* gene in arabidopsis; they are also called sulfurtransferases 1 (*STR1*). Of note, a mutation of *STR1* alone resulted in a shrunken seed phenotype. The shrunken seed phenotype was associated with delayed/arrested embryo development (Mao et al., 2011). In addition, the *MST1* family gene *OsMST5* plays an important role in early seed development in rice (Takeda et al., 2001). *AET5Gv20084100* was flanking SNP marker *F5XZDLF01AU4HH_125* for KW on chromosome 5D. It was homologous to the *ZAR1* gene in arabidopsis, which belongs to the *RLK/Pelle* kinase family (Yu et al., 2016). Maize *ARGOS1* (*ZAR1*) transgenic alleles increase hybrid maize yield, as *ZAR1* increased plant and organ size primarily through increasing cell numbers (Guo et al., 2013). *AET5Gv20005900* was flanking SNP marker *be405667Contig1ATwsnp1* for KV and KSA on chromosome 5D, and it was homologous to *ETR1* in arabidopsis, respectively. In Rice, a reduction of *ETR2* expression could increase the thousand-seed weight (Wuriyanghan et al., 2009). However, enhanced ethylene response also may be related to a larger or heavier kernel (Yin et al., 2017). The starch and protein of rice kernel determine factors of seed dry weight and size (Duan and Sun, 2005; Yin et al., 2017). Thus, *AET5Gv20005900* may presumably affect starch accumulation in *A. tauschii*, finally affecting kernel size. The *AET4Gv20799000* gene related to KW and KWL was predicted on the 4D chromosome. It was homologous to the *MAC3B* gene in arabidopsis, which belongs to the *U-box* family. *U-box* is a ubiquitin ligase activity-related protein domain in plants (Li and Li, 2014; Li S. et al., 2018). Ubiquitin ligases have been identified as key factors of seed size control in plants (Li N. et al., 2018). For example, grain width gene *GW2* encodes a *RING-type E3* ubiquitin ligase and controls kernel width and weight in rice (Matsuoka and Ashikari, 2007). Finally, *AET7Gv20644900* was homologous to *NAC047* in arabidopsis and belonged to the *NAC* gene family. It was reported that *NAC2* regulates embryogenesis, affecting seed shapes in arabidopsis (Kunieda et al., 2008; Mathew et al., 2016). As *NAC2* and *NAC047* belong to the same family, we speculate that these candidate genes may affect embryo development, kernel size, and yield. The genes identified by GWAS can be speeds up selective breeding using *CRISPR-Cas9* system, which is a powerful tool for rapid and effective genetic improvement and allows several QTL/genes to be edited precisely and simultaneously or even novel alleles to be created.

## Conclusion

This in-depth study of *A. tauschii* provides new insight into its potential role in wheat improvement. Six kernel traits, including KL, KW, KWL, KSA, KV, and HKW, were evaluated among 223 *A. tauschii* over 3 years. *H’* was in the range 0.80-0.89, showing that *A. tauschii* had high diversity. Kernel traits showed medium to high heritability (0.74-0.87), and correlation and linear regressions analyses showed that HKW increased with increasing KL, KW, and KV. Kernel size traits affected kernel weight and, subsequently, yield. Our research results revealed that there are favorable varieties with longer and wider kernels in both subspecies of *A. tauschii*. Based on BLUP values, a total of 66 significant SNPs was identified using GWAS, and six candidate genes were identified as potential genetic drivers of these yield-related traits. The identified SNPs/genes will speed up the wheat breeding by MAS and genome-editing technology. It is expected that the excellent target gene from the D subgenome can be successfully introduced into wheat, so as to increase the yield of wheat and broaden the genomic resources of wheat.

## Data Availability Statement

The raw data supporting the conclusions of this article will be made available by the authors, without undue reservation.

## Author Contributions

QW and NY drafted and revised the manuscript and contributed to data analysis. HC, HH, YL, HS, KZ, XJ, and SY performed the phenotypic evaluation and helped with data analysis. SL helped to perform linkage disequilibrium analysis and revise the manuscript. CL, GC, and ZY helped to draft the manuscript. YL designed and coordinated the study and revised the manuscript. All authors have read and approved the final manuscript for publication.

## Conflict of Interest

The authors declare that the research was conducted in the absence of any commercial or financial relationships that could be construed as a potential conflict of interest.

## Footnotes

1 https://www.ebi.ac.uk/ena/browser/view/GCA_002575655.1

## Supplementary Material

Supplementary Figure 1 The distribution of 6723 SNP markers on seven chromosomes of *Aegilops tauschii*.

Supplementary Figure 2 Linkage disequilibrium (LD) decay plot for T-group and S-group.

Supplementary Figure 3 Frequency distribution of six kernel trait in *Aegilops tauschii*.

Supplementary Table 1 Genotype classification, morphological classification and cluster analysis of 223 *Aegilops tauschii* accessions.

Supplementary Table 2 Distribution of SNP markers on chromosomes and polymorphism information content (PIC) of diverse chromosomes.

Supplementary Table 3 Descriptive analysis, coefficient of variation of six kernel traits among environments.

Supplementary Table 4 Comparison of six kernel characters between T-group and S-group.

Supplementary Table 5 Multiple linear stepwise regression to explain hundred-kernel weight (HKW) from other kernel traits based on BLUP values.

Supplementary Table 6 Through genome-wide association analysis of various environmental traits in 223 materials, significant SNPs were identified for kernel traits.

Supplementary Table 7 Genes Homologous annotation using KOBAS 3.0.

Supplementary Table 8 The six kernel traits measured in multiple trials.
