# Supplementary material for: Genome-Wide Association Study of Kernel Traits in Aegilops tauschii
Source: Front Genet. 2021 May 28;12:651785. doi: 10.3389/fgene.2021.651785 (PMC8194309; doi:10.3389/fgene.2021.651785)

Supplementary Fig. S1 The distribution of 6723 SNP markers on seven chromosomes of *Aegilops tauschii.*

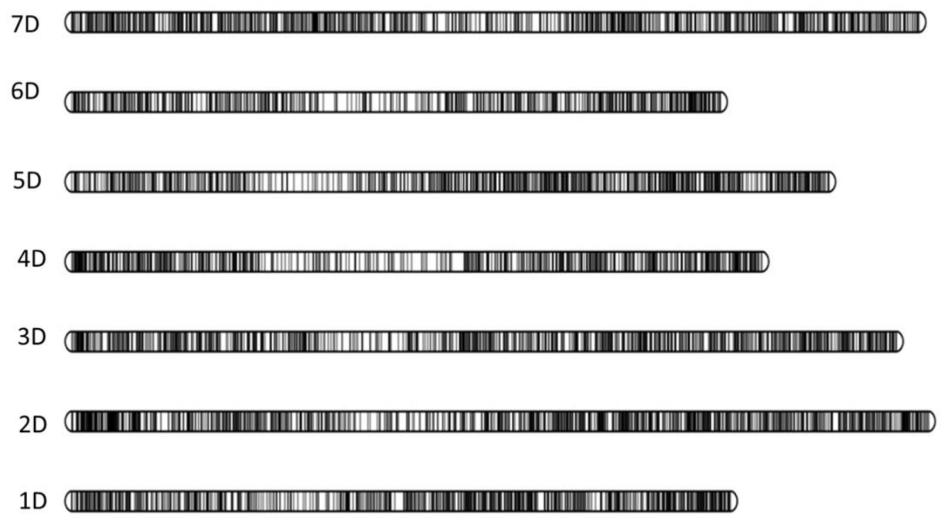

Supplement: Supplementary Figure 1 — The distribution of 6723 SNP markers on seven chromosomes of Aegilops tauschii. [file Image_1.pdf]
