# Supplementary material for: Genome-Wide Association Study of Kernel Traits in Aegilops tauschii
Source: Front Genet. 2021 May 28;12:651785. doi: 10.3389/fgene.2021.651785 (PMC8194309; doi:10.3389/fgene.2021.651785)
Supplement: Supplementary Figure 2 — Linkage disequilibrium (LD) decay plot for T-group and S-group. [file Image_2.pdf]

Supplementary Fig. S2 Linkage disequilibrium (LD) decay plot for T-group and S-group**.**

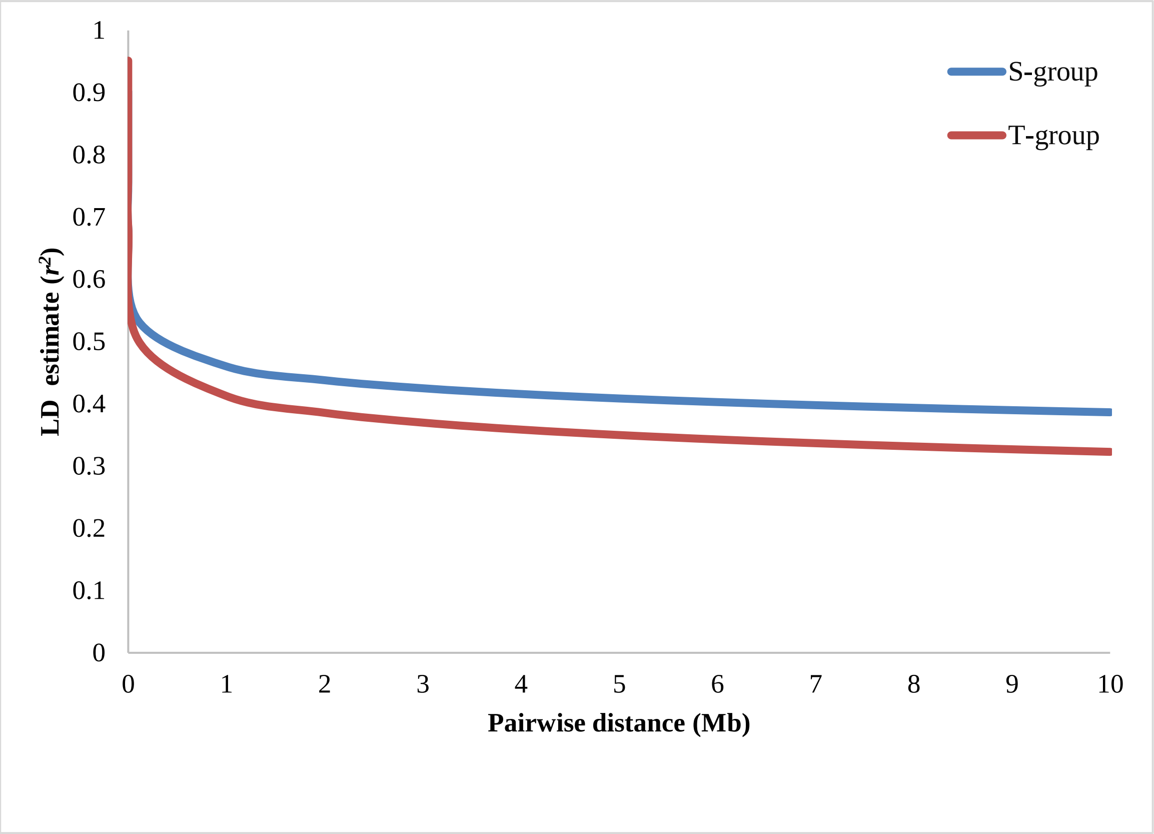

Abbreviation: T-group: A. tauschii ssp. tauschii; S-group: A. tauschii ssp. strangulata.
