# Supplementary material for: Genome-Wide Association Study of Kernel Traits in Aegilops tauschii
Source: Front Genet. 2021 May 28;12:651785. doi: 10.3389/fgene.2021.651785 (PMC8194309; doi:10.3389/fgene.2021.651785)
Supplement: Supplementary Figure 3 — Frequency distribution of six kernel trait in Aegilops tauschii. [file Image_3.pdf]

Supplementary Fig. S3 Frequency distribution of six kernel trait in *Aegilops tauschii*.

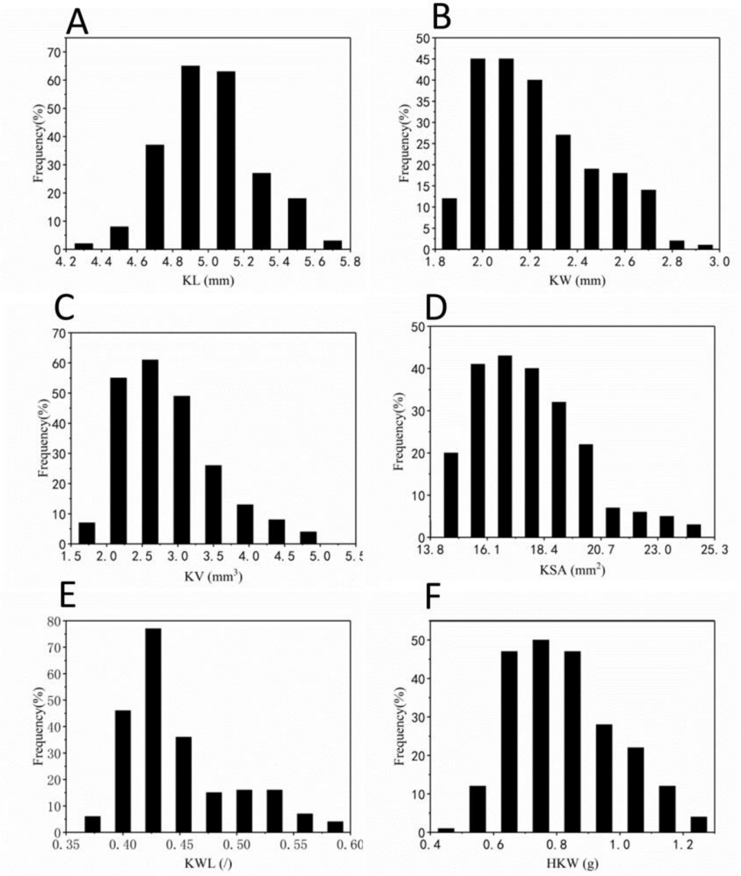

Abbreviation: (A) KL, kernel length, (B) KW, kernel width, (C) KV, kernel volume, (D) KSA, kernel surface area, (E) KWL, kernel width to length ratio, (F) HKW, hundred-kernel weight.
