# Supplementary material for: Genome-Wide Association Study of Kernel Traits in Aegilops tauschii
Source: Front Genet. 2021 May 28;12:651785. doi: 10.3389/fgene.2021.651785 (PMC8194309; doi:10.3389/fgene.2021.651785)
Supplement: Supplementary Table 1 — Genotype classification, morphological classification and cluster analysis of 223 Aegilops tauschii accessions. [file Table_1.docx]

**Supplementary Table S1** Genotype classification, morphological classification and cluster analysis of 223 *Aegilops tauschii* accessions.

| Accession | Origin | Classification based on Zhao et al. 2018 | Classification based on STRUCTURE 2.3.4 | Classification based cluster analysis (Ward's method) |
| --- | --- | --- | --- | --- |
| AS623182 | Unkonwn | *A. strangulata* | T-group | II |
| AS623065 | Afghanistan | *A. tauschii* | T-group | I |
| AS623068 | Afghanistan | *A. tauschii* | T-group | I |
| AS623197 | Afghanistan | *A. tauschii* | T-group | I |
| AS623157 | Armenia/Georgia | *A. tauschii* | T-group | I |
| AS623135 | Afghanistan | *A. tauschii* | T-group | I |
| AS623038 | Unkonwn | *A. tauschii* | T-group | II |
| AS623014 | China | *A. tauschii* | T-group | II |
| AS623020 | China | *A. strangulata* | T-group | I |
| AS623039 | Unkonwn | *A. tauschii* | T-group | I |
| AS623171 | Afghanistan | *A. tauschii* | T-group | I |
| AS623230 | Afghanistan | *A. tauschii* | T-group | I |
| AS623231 | Afghanistan | *A. tauschii* | T-group | I |
| AS623249 | China | *A. tauschii* | T-group | I |
| AS623265 | China | *A. tauschii* | T-group | I |
| AS623008 | Iran | *A. tauschii* | T-group | I |
| AS623071 | Afghanistan | *A. tauschii* | T-group | I |
| AS623023 | China | *A. tauschii* | T-group | I |
| AS623074 | Afghanistan | *A. tauschii* | T-group | I |
| AS623228 | Afghanistan | *A. tauschii* | T-group | I |
| AS623388 | Pakistan | *A. tauschii* | T-group | II |
| AS623189 | Afghanistan | *A. tauschii* | T-group | I |
|  |  |  |  |  |
| AS623369 | Pakistan | *A. tauschii* | T-group | I |
| AS623416 | Tajikistan | *A. tauschii* | T-group | I |
| AS623010 | China | *A. tauschii* | T-group | I |
| AS623060 | Pakistan | *A. tauschii* | T-group | I |
| AS623076 | Afghanistan | *A. tauschii* | T-group | I |
| AS623233 | Kazakhstan | *A. tauschii* | T-group | I |
| AS623236 | Afghanistan | *A. tauschii* | T-group | I |
| AS623237 | Afghanistan | *A. tauschii* | T-group | I |
| AS623268 | Afghanistan | *A. tauschii* | T-group | I |
| AS623386 | Pakistan | *A. tauschii* | T-group | I |
| AS623390 | Pakistan | *A. tauschii* | T-group | I |
| AS623081 | Afghanistan | *A. tauschii* | T-group | I |
| AS623200 | Iran | *A. tauschii* | T-group | I |
| AS623374 | Pakistan | *A. tauschii* | T-group | I |
| AS623379 | Pakistan | *A. tauschii* | T-group | I |
| AS623384 | Pakistan | *A. tauschii* | T-group | I |
| AS623391 | Pakistan | *A. tauschii* | T-group | I |
| AS623392 | Pakistan | *A. tauschii* | T-group | I |
| AS623395 | Pakistan | *A. tauschii* | T-group | I |
| AS623059 | China | *A. tauschii* | T-group | I |
| AS623031 | Unkonwn | *A. tauschii* | T-group | I |
| AS623073 | Afghanistan | *A. tauschii* | T-group | I |
| AS623188 | Afghanistan | *A. tauschii* | T-group | I |
|  |  |  |  |  |
| AS623222 | Unkonwn | *A. tauschii* | T-group | I |
| AS623242 | Turkmenistan | *A. tauschii* | T-group | II |
| AS623399 | Pakistan | *A. tauschii* | T-group | I |
| AS623080 | Afghanistan | *A. tauschii* | T-group | I |
| AS623165 | Pakistan | *A. tauschii* | T-group | I |
| AS623195 | Pakistan | *A. tauschii* | T-group | I |
| AS623270 | Afghanistan | *A. tauschii* | T-group | II |
| AS623368 | Pakistan | *A. tauschii* | T-group | I |
| AS623398 | Pakistan | *A. tauschii* | T-group | I |
| AS623238 | Afghanistan | *A. tauschii* | T-group | I |
| AS623380 | Pakistan | *A. tauschii* | T-group | I |
| AS623383 | Pakistan | *A. tauschii* | T-group | I |
| AS623403 | Pakistan | *A. tauschii* | T-group | I |
| AS623408 | Uzbekistan | *A. tauschii* | T-group | I |
| AS623421 | Uzbekistan | *A. tauschii* | T-group | I |
| AS623422 | Uzbekistan | *A. tauschii* | T-group | I |
| AS623047 | Iran | *A. strangulata* | T-group | II |
| AS623194 | Afghanistan | *A. tauschii* | T-group | I |
| AS623223 | Unkonwn | *A. tauschii* | T-group | I |
| AS623315 | Western Asia | *A. tauschii* | T-group | I |
| AS623396 | Pakistan | *A. tauschii* | T-group | I |
| AS623417 | Uzbekistan | *A. tauschii* | T-group | I |
| AS623002 | France | *A. tauschii* | T-group | I |
|  |  |  |  |  |
| AS623124 | Afghanistan | *A. tauschii* | T-group | I |
| AS623145 | Azerbaijan | *A. tauschii* | T-group | I |
| AS623275 | Unkonwn | *A. tauschii* | T-group | I |
| AS623402 | Pakistan | *A. tauschii* | T-group | I |
| AS623413 | Tajikistan | *A. tauschii* | T-group | I |
| AS623418 | Uzbekistan | *A. tauschii* | T-group | I |
| AS623311 | Russian Federation | *A. tauschii* | T-group | I |
| AS623355 | Uzbekistan | *A. tauschii* | T-group | I |
| AS623378 | Pakistan | *A. tauschii* | T-group | I |
| AS623409 | Uzbekistan | *A. tauschii* | T-group | I |
| AS623312 | Armenia | *A. tauschii* | T-group | I |
| AS623354 | Uzbekistan | *A. tauschii* | T-group | I |
| AS623411 | Uzbekistan | *A. tauschii* | T-group | I |
| AS623057 | Iran | *A. tauschii* | T-group | I |
| AS623064 | Afghanistan | *A. tauschii* | T-group | I |
| AS623133 | Afghanistan | *A. tauschii* | T-group | I |
| AS623363 | Tajikistan | *A. tauschii* | T-group | I |
| AS623362 | Tajikistan | *A. strangulata* | T-group | I |
| AS623414 | Tajikistan | *A. tauschii* | T-group | I |
| AS623181 | Unkonwn | *A. tauschii* | T-group | I |
| AS623198 | Afghanistan | *A. tauschii* | T-group | I |
| AS623304 | Turkey | *A. tauschii* | T-group | I |
| AS623366 | Tajikistan | *A. tauschii* | T-group | I |
|  |  |  |  |  |
| AS623313 | India | *A. strangulata* | T-group | I |
| AS623352 | Uzbekistan | *A. tauschii* | T-group | I |
| AS623003 | Unkonwn | *A. tauschii* | T-group | I |
| AS623146 | Afghanistan | *A. tauschii* | T-group | I |
| AS623161 | Afghanistan | *A. tauschii* | T-group | I |
| AS623303 | Turkey | *A. tauschii* | T-group | I |
| AS623307 | Turkey | *A. tauschii* | T-group | II |
| AS623030 | Unkonwn | *A. strangulata* | T-group | II |
| AS623130 | Afghanistan | *A. strangulata* | T-group | I |
| AS623159 | Georgia | *A. tauschii* | T-group | I |
| AS623253 | Turkey | *A. tauschii* | T-group | I |
| AS623294 | Turkey | *A. tauschii* | T-group | I |
| AS623297 | Turkey | *A. tauschii* | T-group | I |
| AS623357 | Kazakhstan | *A. tauschii* | T-group | I |
| AS623119 | Iran | *A. tauschii* | T-group | I |
| AS623158 | Armenia | *A. tauschii* | T-group | I |
| AS623271 | Afghanistan | *A. tauschii* | T-group | I |
| AS623285 | Turkey | *A. tauschii* | T-group | I |
| AS623287 | Turkey | *A. tauschii* | T-group | I |
| AS623288 | Turkey | *A. tauschii* | T-group | I |
| AS623292 | Turkey | *A. strangulata* | T-group | I |
| AS623258 | Turkey | *A. tauschii* | T-group | I |
| AS623067 | Afghanistan | *A. tauschii* | T-group | I |
|  |  |  |  |  |
| AS623389 | Pakistan | *A. tauschii* | T-group | I |
| AS623005 | Unkonwn | *A. tauschii* | T-group | I |
| AS623370 | Pakistan | *A. tauschii* | T-group | I |
| AS623400 | Pakistan | *A. tauschii* | T-group | I |
| AS623407 | Uzbekistan | *A. tauschii* | T-group | I |
| AS623419 | Uzbekistan | *A. strangulata* | T-group | II |
| AS623296 | Turkey | *A. tauschii* | T-group | I |
| AS623295 | Turkey | *A. tauschii* | T-group | I |
| AS623406 | India | *A. tauschii* | T-group | I |
| AS623349 | Syrian Arab Republic | *A. tauschii* | T-group | I |
| AS623338 | Western Asia | *A. strangulata* | T-group | I |
| AS623186 | Afghanistan | *A. tauschii* | T-group | I |
| AS623501 | Afghanistan | *A. tauschii* | T-group | I |
| AS623503 | Afghanistan | *A. tauschii* | T-group | I |
| AS623508 | Iran | *A. tauschii* | T-group | I |
| AS623286 | Turkey | *A. tauschii* | T-group | I |
| AS623524 | Iran | *A. strangulata* | T-group | I |
| AS623595 | Afghanistan | *A. tauschii* | T-group | I |
| AS623599 | Afghanistan | *A. tauschii* | T-group | I |
| AS623619 | Afghanistan | *A. tauschii* | T-group | I |
| AS623642 | Armenia/Georgia | *A. tauschii* | T-group | I |
| AS623467 | Afghanistan | *A. tauschii* | T-group | I |
| AS623470 | Afghanistan | *A. tauschii* | T-group | I |
|  |  |  |  |  |
| AS623458 | Afghanistan | *A. tauschii* | T-group | I |
| AS623523 | Iran | *A. strangulata* | T-group | II |
| AS623090 | Iran | *A. strangulata* | S-group | II |
| AS623141 | Azerbaijan | *A. strangulata* | S-group | II |
| AS623324 | Azerbaijan | *A. strangulata* | S-group | I |
| AS623325 | Azerbaijan | *A. strangulata* | S-group | I |
| AS623427 | Iran | *A. strangulata* | S-group | II |
| AS623272 | Iran | *A. strangulata* | S-group | II |
| AS623174 | Iran | *A. strangulata* | S-group | II |
| AS623214 | Iran | *A. strangulata* | S-group | II |
| AS623246 | Turkmenistan | *A. strangulata* | S-group | II |
| AS623247 | Azerbaijan | *A. strangulata* | S-group | II |
| AS623117 | Iran | *A. strangulata* | S-group | II |
| AS623193 | Iran | *A. strangulata* | S-group | II |
| AS623024 | China | *A. strangulata* | S-group | II |
| AS623118 | Iran | *A. strangulata* | S-group | II |
| AS623277 | Iran | *A. strangulata* | S-group | II |
| AS623335 | Turkmenistan | *A. tauschii* | S-group | II |
| AS623250 | Turkey | *A. strangulata* | S-group | II |
| AS623216 | Iran | *A. tauschii* | S-group | II |
| AS623211 | Iran | *A. strangulata* | S-group | II |
| AS623251 | Turkey | *A. strangulata* | S-group | II |
| AS623252 | Turkey | *A. strangulata* | S-group | II |
|  |  |  |  |  |
| AS623329 | Turkmenistan | *A. strangulata* | S-group | II |
| AS623034 | Iran | *A. strangulata* | S-group | II |
| AS623206 | Iran | *A. strangulata* | S-group | II |
| AS623212 | Iran | *A. strangulata* | S-group | II |
| AS623243 | Azerbaijan | *A. strangulata* | S-group | II |
| AS623339 | Iran | *A. strangulata* | S-group | II |
| AS623127 | Azerbaijan | *A. strangulata* | S-group | I |
| AS623170 | Unkonwn | *A. strangulata* | S-group | II |
| AS623205 | Iran | *A. strangulata* | S-group | II |
| AS623210 | Iran | *A. strangulata* | S-group | I |
| AS623226 | Unkonwn | *A. strangulata* | S-group | II |
| AS623240 | Unkonwn | *A. strangulata* | S-group | II |
| AS623280 | Iran | *A. strangulata* | S-group | II |
| AS623306 | Turkey | *A. strangulata* | S-group | II |
| AS623051 | Turkmenistan | *A. tauschii* | S-group | I |
| AS623166 | Unkonwn | *A. strangulata* | S-group | I |
| AS623209 | Iran | *A. strangulata* | S-group | I |
| AS623239 | Azerbaijan | *A. strangulata* | S-group | II |
| AS623244 | Russian Federation | *A. strangulata* | S-group | I |
| AS623326 | Azerbaijan | *A. strangulata* | S-group | II |
| AS623040 | Iran | *A. strangulata* | S-group | I |
| AS623048 | Iran | *A. strangulata* | S-group | II |
| AS623125 | Azerbaijan | *A. tauschii* | S-group | I |
|  |  |  |  |  |
| AS623279 | Iran | *A. strangulata* | S-group | II |
| AS623322 | Azerbaijan | *A. strangulata* | S-group | I |
| AS623343 | Iran | *A. tauschii* | S-group | I |
| AS623346 | Azerbaijan | *A. strangulata* | S-group | II |
| AS623183 | Iran | *A. strangulata* | S-group | II |
| AS623201 | Iran | *A. strangulata* | S-group | I |
| AS623044 | Unkonwn | *A. strangulata* | S-group | II |
| AS623046 | Iran | *A. strangulata* | S-group | II |
| AS623177 | Azerbaijan | *A. strangulata* | S-group | II |
| AS623330 | Azerbaijan | *A. strangulata* | S-group | I |
| AS623333 | Azerbaijan | *A. strangulata* | S-group | II |
| AS623037 | Unkonwn | *A. strangulata* | S-group | I |
| AS623045 | Unkonwn | *A. strangulata* | S-group | II |
| AS623054 | Armenia | *A. strangulata* | S-group | II |
| AS623136 | Afghanistan | *A. strangulata* | S-group | II |
| AS623150 | Azerbaijan | *A. strangulata* | S-group | I |
| AS623168 | Unkonwn | *A. strangulata* | S-group | I |
| AS623241 | Azerbaijan | *A. strangulata* | S-group | II |
| AS623319 | Iran | *A. strangulata* | S-group | I |
| AS623332 | Azerbaijan | *A. strangulata* | S-group | II |
| AS623007 | Unkonwn | *A. strangulata* | S-group | II |
| AS623137 | Azerbaijan | *A. strangulata* | S-group | II |
| AS623167 | Azerbaijan | *A. tauschii* | S-group | II |
|  |  |  |  |  |
| AS623232 | Iran | *A. strangulata* | S-group | II |
| AS623282 | Turkey | *A. tauschii* | S-group | II |
| AS623320 | Iran | *A. strangulata* | S-group | I |
| AS623203 | Iran | *A. strangulata* | S-group | I |
| AS623281 | Iran | *A. strangulata* | S-group | I |
| AS623142 | Azerbaijan | *A. strangulata* | S-group | I |
| AS623043 | Unkonwn | *A. strangulata* | S-group | II |
| AS623327 | Azerbaijan | *A. strangulata* | S-group | II |
| AS623274 | Iran | *A. strangulata* | S-group | II |
| AS623438 | Iran | *A. strangulata* | S-group | I |
| AS623202 | Iran | *A. strangulata* | S-group | I |
| AS623525 | Iran | *A. tauschii* | S-group | II |
| AS623534 | Iran | *A. strangulata* | S-group | I |
| AS623535 | Iran | *A. strangulata* | S-group | I |
| AS623540 | Iran | *A. strangulata* | S-group | II |
| AS623629 | Armenia | *A. strangulata* | S-group | II |
| AS623522 | Iran | *A. strangulata* | S-group | II |

Reference: Zhao, L., Ning, S., Yi, Y., Zhang, L., Yuan, Z., Wang, J., et al. (2018). Fluorescence in situ hybridization karyotyping reveals the resence of two distinct genomes in the taxon *Aegilops* *tauschii*. *BMC Genomics 19*. https://doi.org/10.1186/s12864-017-4384-0
