# Supplementary material for: Genome-Wide Association Study of Kernel Traits in Aegilops tauschii
Source: Front Genet. 2021 May 28;12:651785. doi: 10.3389/fgene.2021.651785 (PMC8194309; doi:10.3389/fgene.2021.651785)
Supplement: Supplementary Table 2 — Distribution of SNP markers on chromosomes and polymorphism information content (PIC) of diverse chromosomes. [file Table_2.docx]

| **Supplementary Table S2** Distribution of SNP markers on chromosomes and polymorphism information content (PIC) of diverse chromosomes. | | | | | | |
| --- | --- | --- | --- | --- | --- | --- |
|  |  |  |  |  |  |  |
| Chromosome | SNP number | Map length (Mb) | Marker density (Mb/per SNP) | Min PIC | Max PIC | Mean PIC |
| 1D | 896 | 502.18 | 0.56 | 0.10 | 0.50 | 0.41 |
| 2D | 1231 | 651.08 | 0.53 | 0.10 | 0.50 | 0.42 |
| 3D | 1029 | 626.33 | 0.61 | 0.10 | 0.50 | 0.43 |
| 4D | 784 | 525.77 | 0.67 | 0.10 | 0.50 | 0.44 |
| 5D | 969 | 576.73 | 0.60 | 0.10 | 0.50 | 0.41 |
| 6D | 711 | 495.52 | 0.70 | 0.10 | 0.50 | 0.42 |
| 7D | 1103 | 644.57 | 0.58 | 0.10 | 0.50 | 0.42 |
| All | 6723 | 4022.18 | 0.61 | 0.10 | 0.50 | 0.42 |
