# Supplementary material for: Genome-Wide Association Study of Kernel Traits in Aegilops tauschii
Source: Front Genet. 2021 May 28;12:651785. doi: 10.3389/fgene.2021.651785 (PMC8194309; doi:10.3389/fgene.2021.651785)
Supplement: Supplementary Table 3 — Descriptive analysis, coefficient of variation of six kernel traits among environments. [file Table_3.docx]

**Supplementary Table S3** Descriptive analysis, coefficient of variation of six kernel traits among environments.

| Trait | Mean ± SD | | | CV (%) | | | Min | | | Max | | |
| --- | --- | --- | --- | --- | --- | --- | --- | --- | --- | --- | --- | --- |
|  | 2017 | 2018 | 2019 | 2017 | 2018 | 2019 | 2017 | 2018 | 2019 | 2017 | 2018 | 2019 |
| KL (mm) | 5.14±0.45 | 5.19±0.44 | 4.72±0.40 | 8.81 | 8.51 | 8.49 | 4.03 | 4.14 | 3.79 | 6.87 | 6.71 | 5.94 |
| KW (mm) | 1.96±0.31 | 2.41±0.31 | 2.32±0.30 | 15.70 | 12.82 | 13.15 | 1.04 | 1.80 | 1.84 | 2.97 | 3.24 | 3.14 |
| KV (mm^3^) | 1.53±0.75 | 3.89±1.15 | 3.22±1.04 | 48.99 | 29.47 | 32.22 | 0.60 | 1.83 | 1.72 | 5.50 | 7.50 | 6.67 |
| KSA (mm^2^) | 12.41±3.26 | 21.91±3.74 | 18.97±3.49 | 26.31 | 17.07 | 18.42 | 6.60 | 14.31 | 13.28 | 26.75 | 32.91 | 30.30 |
| KWL (/) | 0.38±0.06 | 0.47±0.06 | 0.49±0.06 | 16.87 | 13.45 | 12.99 | 0.21 | 0.36 | 0.38 | 0.61 | 0.67 | 0.68 |
| HKW (g) | / | 0.92±0.28 | 0.73±0.27 | / | 30.52 | 37.59 | / | 0.26 | 0.21 | / | 1.77 | 1.50 |

Abbreviation: SD, standard deviation; CV, coefficient of variation; KL, kernel length; KW, kernel width; KV, kernel volume; KSA, kernel surface area; KWL, kernel width to length ratio; HKW, hundred-kernel weight.
