# Supplementary material for: Genome-Wide Association Study of Kernel Traits in Aegilops tauschii
Source: Front Genet. 2021 May 28;12:651785. doi: 10.3389/fgene.2021.651785 (PMC8194309; doi:10.3389/fgene.2021.651785)
Supplement: Supplementary Table 4 — Comparison of six kernel characters between T-group and S-group. [file Table_4.docx]

| **Supplementary Table S4** Comparison of six kernel characters between T-group and S-group. | | | | | | | |
| --- | --- | --- | --- | --- | --- | --- | --- |
| Trait | KL (mm) | KW (mm) | KV (mm^3^) | KSA (mm^2^) | KWL (/) | HKW (g) |  |
| T-group | | | | | | |  |
| Min | 4.31 | 1.83 | 1.84 | 13.71 | 0.37 | 0.49 |  |
| Max | 5.51 | 2.67 | 4.57 | 23.02 | 0.54 | 1.26 |  |
| Mean | 4.97 | 2.09 | 2.51 | 16.56 | 0.43 | 0.75 |  |
| SD | 0.22 | 0.15 | 0.41 | 1.50 | 0.03 | 0.13 |  |
| CV (%) | 4.34 | 7.11 | 16.45 | 9.07 | 6.87 | 17.36 |  |
| S-group | | | | | | |  |
| Min | 4.45 | 2.07 | 2.35 | 16.01 | 0.39 | 0.61 |  |
| Max | 5.77 | 2.89 | 5.01 | 24.81 | 0.59 | 1.27 |  |
| Mean | 5.09 | 2.45 | 3.48 | 19.70 | 0.48 | 0.95 |  |
| SD | 0.30 | 0.19 | 0.59 | 1.95 | 0.05 | 0.14 |  |
| CV (%) | 5.94 | 7.59 | 17.03 | 9.88 | 10.40 | 15.02 |  |

Abbreviation: CV, coefficient of variation; KL, kernel length; KW, kernel width; KV, kernel volume; KSA, kernel surface area;

KWL, kernel width to length ratio; HKW, hundred-kernel weight; SD, standard deviation.
