# Supplementary material for: Genome-Wide Association Study of Kernel Traits in Aegilops tauschii
Source: Front Genet. 2021 May 28;12:651785. doi: 10.3389/fgene.2021.651785 (PMC8194309; doi:10.3389/fgene.2021.651785)
Supplement: Supplementary Table 5 — Multiple linear stepwise regression to explain hundred-kernel weight (HKW) from other kernel traits based on BLUP values. [file Table_5.docx]

| **Supplementary Table S5** Multiple linear stepwise regression to explain hundred-kernel weight (HKW) from other kernel traits based on BLUP values. | | | | |
| --- | --- | --- | --- | --- |
|  |  |  |  |  |
| Trait | Final stepwise model | R^2^ | P value |  |
| HKW | -0.968+0.302*KV+0.327*KL-0.078KSA+0.298KW | 0.768 | <0.001 |  |

Abbreviation: KL, kernel length; KW, kernel width; KV, kernel volume; KSA, kernel surface area; HKW, hundred-kernel weight, R^2^: phenotypic variation explained by the final stepwise model.
