# Supplementary material for: Genome-Wide Association Study of Kernel Traits in Aegilops tauschii
Source: Front Genet. 2021 May 28;12:651785. doi: 10.3389/fgene.2021.651785 (PMC8194309; doi:10.3389/fgene.2021.651785)
Supplement: Supplementary Table 6 — Through genome-wide association analysis of various environmental traits in 223 materials, significant SNPs were identified for kernel traits. [file Table_6.docx]

**Supplementary Table S6** Through genome-wide association analysis of various environmental traits in 223 materials, significant SNPs were identified for kernel traits

| Marker | Trait | Chr | Position (Mb) | -log_10_*^(p)^* | | | | PVE (%) | | | |
| --- | --- | --- | --- | --- | --- | --- | --- | --- | --- | --- | --- |
|  |  |  |  | 2017 | 2018 | 2019 | BLUP | 2017 | 2018 | 2019 | BLUP |
| *GBUVHFX02HVP40-311* | KV | 1D | 116.88 | 3.36 |  |  |  | 5.48 |  |  |  |
| *GBF1XID01AROUO-311* | KV | 1D | 122.84 | 3.36 |  |  |  | 5.48 |  |  |  |
| *GBB4FNX02HYMF8-129* | KV | 1D | 122.86 | 3.36 |  |  |  | 5.48 |  |  |  |
| *GBF1XID01C2C4J-80* | KV | 1D | 135.03 | 3.36 |  |  |  | 5.48 |  |  |  |
| *BF429033-ATwsnp1* | KV | 1D | 136.02 | 3.36 |  |  |  | 5.48 |  |  |  |
| *F5XZDLF02ICENN-280* | KV | 1D | 140.92 | 3.36 |  |  |  | 5.48 |  |  |  |
| *GCE8AKX01CB8RX-268* | KV | 1D | 140.92 | 3.36 |  |  |  | 5.48 |  |  |  |
| *GA8KES401ER1G2-419* | KV | 1D | 151.78 | 3.36 |  |  |  | 5.48 |  |  |  |
| *GCE8AKX02IOYE5-131* | KV | 1D | 153.42 | 3.36 |  |  |  | 5.48 |  |  |  |
| *F5MV3MU01B0058-94* | KV | 1D | 165.20 | 3.36 |  |  |  | 5.48 |  |  |  |
| *F1BEJMU01DRL19-157* | KV | 1D | 178.36 | 3.35 |  |  |  | 5.83 |  |  |  |
| *GDS7LZN02J2U9I-149* | KV | 1D | 193.82 | 3.36 |  |  |  | 5.48 |  |  |  |
| *GA8KES401AZZ81-419* | KV | 1D | 195.03 | 3.36 |  |  |  | 5.48 |  |  |  |
| *GDEEGVY02GVTQG-115* | KL | 1D | 302.59 |  |  |  | 3.07 |  |  |  | 4.94 |
| *GBUVHFX01COQEJ-254* | KV | 1D | 307.43 | 3.31 |  |  |  | 5.39 |  |  |  |
| *F1BEJMU02HVVCO-182* | KSA | 1D | 337.46 |  | 3.81 |  |  |  | 6.32 |  |  |
|  | KV | 1D | 337.46 |  | 3.73 |  |  |  | 6.15 |  |  |
| *GDS7LZN02GQ7FT-204* | KSA | 1D | 338.28 |  | 4.59 |  |  |  | 7.76 |  |  |
|  | KL | 1D | 338.28 |  | 4.38 |  | 3.37 |  | 7.38 |  | 5.50 |
|  | KV | 1D | 338.28 |  | 3.58 |  |  |  | 5.88 |  |  |
|  | HKW | 1D | 338.28 |  | 3.01 |  |  |  | 4.84 |  |  |
| *GA8KES401AX4UZ-33* | KL | 1D | 366.14 |  | 3.68 |  |  |  | 6.09 |  |  |
|  |  |  |  |  |  |  |  |  |  |  |  |
| *GB5Y7FA01BZBJQ-43* | KWL | 1D | 385.61 |  |  | 4.05 | 3.10 |  |  | 6.78 | 4.98 |
|  | KL | 1D | 385.61 |  |  |  | 3.63 |  |  |  | 5.98 |
| *GB5Y7FA01DQU4S-203* | KV | 1D | 451.90 |  |  | 3.03 |  |  |  | 4.88 |  |
| *GDS7LZN01DH431-386* | KSA | 1D | 453.46 | 4.42 |  |  |  | 7.40 |  |  |  |
|  | KV | 1D | 453.46 | 3.96 |  |  |  | 6.58 |  |  |  |
| *F5XZDLF01DA5XW-133* | KSA | 1D | 457.02 | 5.25 |  |  |  | 8.91 |  |  |  |
|  | KV | 1D | 457.02 | 5.71 |  |  |  | 9.78 |  |  |  |
|  | KW | 1D | 457.02 | 4.06 |  |  |  | 6.74 |  |  |  |
| *F5XZDLF01DJ2ED-256* | KL | 1D | 486.10 |  | 3.09 |  |  |  | 4.99 |  |  |
| *GDS7LZN01EX3OX-40* | KL | 2D | 30.25 | 3.68 |  |  |  | 6.10 |  |  |  |
| *F5XZDLF01CA9QM-167* | KWL | 2D | 30.74 |  |  | 3.72 |  |  |  | 6.16 |  |
|  | KW | 2D | 30.74 |  |  | 3.16 |  |  |  | 5.12 |  |
| *contig06292-134* | KSA | 2D | 355.54 | 4.58 |  |  |  | 7.69 |  |  |  |
|  | KV | 2D | 355.54 | 3.69 |  |  |  | 6.09 |  |  |  |
| *GDS7LZN01CJVSE-167* | KV | 2D | 359.75 | 3.55 |  |  |  | 5.83 |  |  |  |
| *GBB4FNX02HPKLK-84* | KW | 2D | 380.90 | 4.24 |  |  |  | 7.06 |  |  |  |
|  | KV | 2D | 380.90 |  |  |  | 3.28 |  |  |  | 5.31 |
|  | KSA | 2D | 380.90 |  |  |  | 3.05 |  |  |  | 4.87 |
| *F1BEJMU02GDKR8-169* | KWL | 2D | 389.17 |  |  | 4.05 | 3.10 |  |  | 6.78 | 4.98 |
|  | KL | 2D | 389.17 |  |  |  | 3.63 |  |  |  | 5.98 |
| *GB5Y7FA02HYWFA-181* | KW | 2D | 434.39 | 4.31 |  |  | 3.19 | 7.17 |  |  | 5.14 |
| *F5MV3MU01BWFSV-121* | KW | 2D | 436.83 | 4.31 |  |  | 3.19 | 7.17 |  |  | 5.14 |
|  |  |  |  |  |  |  |  |  |  |  |  |
| *GBF1XID01D2CAC-283* | KW | 2D | 438.95 | 4.31 |  |  | 3.19 | 7.17 |  |  | 5.14 |
| *GDS7LZN02F3F6D-133* | KW | 2D | 438.95 | 4.31 |  |  | 3.19 | 7.17 |  |  | 5.14 |
| *contig00547-73* | KW | 2D | 438.99 | 4.68 |  |  | 3.54 | 7.95 |  |  | 5.86 |
|  | KV | 2D | 438.99 |  |  |  | 3.13 |  |  |  | 5.11 |
| *GBF1XID01BTR1G-231* | KW | 2D | 439.88 | 4.31 |  |  | 3.19 | 7.17 |  |  | 5.14 |
| *GBB4FNX02F7Z6S-223* | KW | 2D | 439.88 | 4.31 |  |  | 3.19 | 7.17 |  |  | 5.14 |
| *contig02254-138* | KW | 2D | 441.41 | 4.31 |  |  | 3.19 | 7.17 |  |  | 5.14 |
| *GB5Y7FA01AVQF0-304* | KW | 2D | 452.91 | 3.23 |  |  |  | 5.20 |  |  |  |
| *contig74282-300* | KV | 2D | 523.74 | 3.05 |  |  | 3.38 | 4.91 |  |  | 5.48 |
|  | KW | 2D | 523.74 | 4.96 |  |  |  | 8.35 |  |  |  |
|  | KSA | 2D | 523.74 |  |  |  | 3.27 |  |  |  | 5.29 |
| *contig26616-488* | KWL | 2D | 530.16 |  | 3.01 |  |  |  | 4.85 |  |  |
| *GBF1XID01A7V8B-200* | HKW | 2D | 591.33 |  |  | 3.96 |  |  |  | 6.61 |  |
|  | KV | 2D | 591.33 |  |  | 3.63 |  |  |  | 6.00 |  |
|  | KW | 2D | 591.33 |  |  | 3.42 |  |  |  | 5.60 |  |
| *GCE8AKX01CEXH9-109* | KL | 3D | 8.14 |  | 3.36 |  |  |  | 5.63 |  |  |
| *contig30400-369* | KV | 3D | 9.17 |  |  | 3.34 |  |  |  | 5.52 |  |
| *GBUVHFX02IS590-241* | KV | 3D | 12.03 | 3.33 |  |  |  | 5.43 |  |  |  |
| *GDEEGVY02GT0DS-121* | KV | 3D | 12.07 | 3.32 |  |  |  | 5.50 |  |  |  |
| *GBUVHFX02G2BTK-399* | HKW | 3D | 74.74 |  |  |  | 3.03 |  |  |  | 4.86 |
| *BE446087-ATwsnp2* | HKW | 3D | 77.28 |  |  |  | 3.03 |  |  |  | 4.86 |
| *GBB4FNX02HGIDA-327* | KWL | 3D | 220.32 |  | 3.27 |  |  |  | 5.32 |  |  |
| *GA8KES401DLK10-307* | KWL | 3D | 255.65 |  | 3.28 |  |  |  | 5.36 |  |  |
|  |  |  |  |  |  |  |  |  |  |  |  |
| *GDS7LZN02FY2TV-84* | KWL | 3D | 516.34 | 3.04 |  |  |  | 4.90 |  |  |  |
| *GBUVHFX02H7US2-55* | KW | 3D | 545.85 |  |  | 4.08 |  |  |  | 6.82 |  |
|  | KV | 3D | 545.85 |  |  | 3.57 |  |  |  | 5.87 |  |
|  | KWL | 3D | 545.85 |  |  | 3.46 |  |  |  | 5.68 |  |
| *GA8KES402GL5A8-239* | KWL | 3D | 561.81 | 4.17 | 4.35 |  | 3.29 | 6.97 | 7.31 |  | 5.34 |
| *GA8KES402H4J28-317* | KV | 3D | 592.84 |  |  | 3.50 |  |  |  | 5.75 |  |
|  | HKW | 3D | 592.84 |  |  | 3.40 |  |  |  | 5.56 |  |
| *contig24884-83* | KV | 3D | 605.26 | 3.04 |  |  |  | 4.89 |  |  |  |
|  | KW | 3D | 605.26 | 3.49 |  |  | 3.23 | 5.69 |  |  | 5.21 |
| *GA8KES402FKCIT-90* | KW | 3D | 605.54 | 3.29 |  |  | 3.49 | 5.78 |  |  | 6.17 |
| *GBB4FNX02JQNSU-161* | HKW | 4D | 14.34 |  | 3.54 |  |  |  | 5.80 |  |  |
| *contig16682-265* | HKW | 4D | 332.74 |  |  | 3.49 | 3.16 |  |  | 5.73 | 5.10 |
| *contig28251-230* | KSA | 4D | 366.17 | 3.83 |  | 4.35 | 5.43 | 6.35 |  | 7.35 | 9.26 |
|  | KV | 4D | 366.17 | 3.07 |  | 5.35 | 5.83 | 4.97 |  | 9.19 | 9.99 |
|  | KW | 4D | 366.17 |  |  | 3.31 | 3.69 |  |  | 5.42 | 6.07 |
| *F1BEJMU01CNNGZ-79* | KWL | 4D | 453.78 |  | 3.55 | 4.97 | 3.95 |  | 5.83 | 8.47 | 6.56 |
|  | KW | 4D | 453.78 |  |  | 5.66 | 4.25 |  |  | 9.71 | 7.07 |
|  | KV | 4D | 453.78 |  |  | 4.22 | 3.12 |  |  | 7.07 | 5.01 |
| *GDEEGVY01C7BQU-446* | KWL | 4D | 461.90 |  |  |  | 3.57 |  |  |  | 5.85 |
| *GDEEGVY01DR0E5-247* | KWL | 4D | 464.90 |  | 3.26 |  | 3.13 |  | 5.31 |  | 5.05 |
| *contig26107-272* | KWL | 4D | 501.56 |  |  | 3.26 |  |  |  | 5.31 |  |
| *GA8KES401CWBR7-178* | KW | 4D | 501.57 | 3.03 |  | 4.19 | 4.03 | 5.03 |  | 7.34 | 6.99 |
|  | KWL | 4D | 501.57 |  | 3.87 | 5.39 | 4.31 |  | 6.73 | 9.67 | 7.59 |
|  |  |  |  |  |  |  |  |  |  |  |  |
| *F5XZDLF01DBHUA-161* | KV | 4D | 503.03 |  |  |  | 3.54 |  |  |  | 5.79 |
|  | KW | 4D | 503.03 |  |  |  | 3.23 |  |  |  | 5.21 |
| *GDS7LZN01CH8YS-326* | KW | 4D | 517.57 | 3.39 | 4.74 | 3.33 | 5.16 | 5.50 | 8.00 | 5.43 | 8.73 |
|  | KWL | 4D | 517.57 | 3.21 | 5.34 | 3.38 | 5.05 | 5.22 | 9.13 | 5.53 | 8.58 |
|  | KV | 4D | 517.57 |  |  |  | 3.97 |  |  |  | 6.56 |
| *GDEEGVY02FLOCP-398* | KWL | 4D | 518.68 |  | 4.85 | 3.72 | 4.77 |  | 8.23 | 6.16 | 8.05 |
|  | KW | 4D | 518.68 |  | 3.13 |  |  |  | 5.05 |  |  |
| *contig26842-436* | KWL | 4D | 519.72 |  | 3.18 |  |  |  | 6.37 |  |  |
| *BE405667-Contig1ATwsnp1* | KV | 5D | 2.89 | 3.06 |  |  | 3.09 | 4.93 |  |  | 4.96 |
|  | KSA | 5D | 2.89 |  |  |  | 3.21 |  |  |  | 5.18 |
| *F5XZDLF01AU4HH-125* | KW | 5D | 32.51 |  |  | 3.63 | 3.03 |  |  | 5.99 | 4.83 |
| *GCE8AKX01A0GWJ-46* | KSA | 5D | 40.74 | 4.91 |  |  |  | 8.63 |  |  |  |
|  | KV | 5D | 40.74 | 7.38 |  |  |  | 13.24 |  |  |  |
| *contig23016-198* | KSA | 5D | 51.94 | 4.66 |  |  |  | 7.83 |  |  |  |
|  | KV | 5D | 51.94 | 4.89 |  |  |  | 8.27 |  |  |  |
|  | KW | 5D | 51.94 | 3.19 |  |  |  | 5.14 |  |  |  |
| *contig04657-38* | KSA | 5D | 62.76 |  | 3.70 |  | 3.30 |  | 6.14 |  | 5.36 |
|  | KV | 5D | 62.76 |  | 3.59 |  | 3.41 |  | 5.93 |  | 5.57 |
|  | HKW | 5D | 62.76 |  |  |  | 3.04 |  |  |  | 4.90 |
| *GCE8AKX02I9OE1-117* | KWL | 5D | 284.72 | 3.11 | 3.78 |  |  | 5.01 | 6.27 |  |  |
| *F5XZDLF02F2TKA-173* | KWL | 5D | 287.43 | 3.11 | 3.78 |  |  | 5.01 | 6.27 |  |  |
| *F1BEJMU01EIYUC-56* | KV | 5D | 358.01 | 4.95 |  |  |  | 8.41 |  |  |  |
| *GB5Y7FA02FN8OZ-253* | KV | 5D | 358.72 |  |  | 3.29 |  |  |  | 5.36 |  |
|  |  |  |  |  |  |  |  |  |  |  |  |
| *GA8KES402HYT7F-263* | KSA | 5D | 369.25 | 3.00 |  |  |  | 4.82 |  |  |  |
|  | KV | 5D | 369.25 | 3.33 |  |  |  | 5.42 |  |  |  |
| *GBB4FNX02IY98G-38* | KWL | 5D | 444.83 |  |  | 3.25 |  |  |  | 5.29 |  |
| *GDS7LZN02GGYM8-194* | KSA | 5D | 451.00 |  |  |  | 3.22 |  |  |  | 5.18 |
| *GDS7LZN02H9RBD-193* | KW | 5D | 506.55 | 3.39 |  | 3.41 | 3.60 | 5.49 |  | 5.58 | 5.87 |
|  | KV | 5D | 506.55 |  |  | 3.62 | 3.31 |  |  | 5.97 | 5.35 |
| *GDRF1KQ02I5OF1-65* | KWL | 5D | 506.76 |  | 3.36 |  |  |  | 5.48 |  |  |
|  | KV | 5D | 506.76 |  |  | 3.98 | 3.30 |  |  | 6.63 | 5.34 |
|  | KW | 5D | 506.76 |  |  | 3.77 | 3.45 |  |  | 6.24 | 5.60 |
| *GDS7LZN02FNEPK-214* | KV | 5D | 508.00 |  | 3.28 |  |  |  | 5.32 |  |  |
|  | KW | 5D | 508.00 |  | 3.06 |  |  |  | 4.91 |  |  |
| *GBB4FNX02GANP9-60* | KV | 5D | 508.00 |  | 3.28 |  |  |  | 5.32 |  |  |
|  | KW | 5D | 508.00 |  | 3.06 |  |  |  | 4.91 |  |  |
| *GDRF1KQ02F8V30-278* | KW | 5D | 508.05 | 4.63 |  | 4.17 | 4.81 | 7.74 |  | 6.98 | 8.09 |
|  | KWL | 5D | 508.05 | 4.27 | 5.04 | 4.29 | 5.48 | 7.16 | 8.59 | 7.21 | 9.37 |
|  | KV | 5D | 508.05 |  |  | 3.36 | 3.02 |  |  | 5.49 | 4.82 |
| *GDS7LZN02FRZ4J-84* | KV | 5D | 508.09 |  | 3.28 |  |  |  | 5.32 |  |  |
|  | KW | 5D | 508.09 |  | 3.06 |  |  |  | 4.91 |  |  |
| *contig00387-688* | KV | 5D | 522.27 | 3.93 |  |  |  | 6.53 |  |  |  |
| *GB5Y7FA01DVFU5-158* | KV | 5D | 524.99 | 3.66 |  |  |  | 6.04 |  |  |  |
| *contig17143-54* | KSA | 5D | 538.15 | 4.85 | 3.19 | 5.06 | 6.83 | 9.24 | 5.93 | 9.85 | 13.36 |
|  | KV | 5D | 538.15 | 5.45 | 3.48 | 5.00 | 6.64 | 10.58 | 6.53 | 9.74 | 13.02 |
|  | HKW | 5D | 538.15 |  |  | 4.37 | 3.74 |  |  | 8.42 | 7.05 |
|  |  |  |  |  |  |  |  |  |  |  |  |
|  | KW | 5D | 538.15 |  |  |  | 3.01 |  |  |  | 5.51 |
| *contig69521-504* | KSA | 5D | 554.40 | 4.90 |  |  |  | 8.32 |  |  |  |
|  | KV | 5D | 554.40 | 7.41 |  |  |  | 12.85 |  |  |  |
| *contig07404-178* | KV | 6D | 3.15 | 3.14 |  |  |  | 5.10 |  |  |  |
| *wsnpbe426362_Contig1_1* | KWL | 6D | 11.02 |  |  |  | 3.05 |  |  |  | 4.90 |
| *contig36144-162* | KWL | 6D | 52.93 |  | 3.51 |  | 3.16 |  | 5.77 |  | 5.10 |
|  | KL | 6D | 52.93 |  | 3.11 |  |  |  | 5.03 |  |  |
| *GBUVHFX01A4N19-108* | HKW | 6D | 300.96 |  |  | 4.20 | 3.41 |  |  | 7.05 | 5.56 |
|  | KSA | 6D | 300.96 |  |  | 3.78 |  |  |  | 6.27 |  |
|  | KL | 6D | 300.96 |  |  | 3.40 |  |  |  | 5.58 |  |
| *BE495949-ATwsnp2* | HKW | 6D | 301.93 |  |  | 4.20 | 3.41 |  |  | 7.05 | 5.56 |
|  | KSA | 6D | 301.93 |  |  | 3.78 |  |  |  | 6.27 |  |
|  | KL | 6D | 301.93 |  |  | 3.40 |  |  |  | 5.58 |  |
| *GBUVHFX01C33YQ-452* | HKW | 6D | 311.94 |  |  | 4.20 | 3.41 |  |  | 7.05 | 5.56 |
|  | KSA | 6D | 311.94 |  |  | 3.78 |  |  |  | 6.27 |  |
|  | KL | 6D | 311.94 |  |  | 3.40 |  |  |  | 5.58 |  |
| *GDS7LZN01EQOYW-155* | HKW | 6D | 313.13 |  |  | 4.20 | 3.41 |  |  | 7.05 | 5.56 |
|  | KSA | 6D | 313.13 |  |  | 3.78 |  |  |  | 6.27 |  |
|  | KL | 6D | 313.13 |  |  | 3.40 |  |  |  | 5.58 |  |
| *contig67633-66* | KWL | 6D | 406.04 |  | 4.30 | 5.36 | 5.65 |  | 7.23 | 9.19 | 9.66 |
| *GBQ4KXB02HX7L4-164* | KWL | 6D | 408.11 |  | 4.30 | 5.36 | 5.65 |  | 7.23 | 9.19 | 9.66 |
| *GBUVHFX01CT9JW-119* | KWL | 6D | 408.11 |  | 4.30 | 5.36 | 5.65 |  | 7.23 | 9.19 | 9.66 |
| *F5XZDLF02JLKIX-54* | KWL | 6D | 408.11 |  | 4.30 | 5.36 | 5.65 |  | 7.23 | 9.19 | 9.66 |
|  |  |  |  |  |  |  |  |  |  |  |  |
| *GBUVHFX01CTPQ5-363* | KW | 7D | 8.27 | 3.26 |  |  |  | 5.49 |  |  |  |
| *contig76625-350* | KWL | 7D | 30.24 |  |  |  | 3.76 |  |  |  | 6.21 |
|  | KL | 7D | 30.24 |  |  |  | 3.39 |  |  |  | 5.54 |
| *contig38516-330* | KWL | 7D | 30.26 |  |  |  | 3.76 |  |  |  | 6.21 |
|  | KL | 7D | 30.26 |  |  |  | 3.39 |  |  |  | 5.54 |
| *GDEEGVY01EZTS0-171* | HKW | 7D | 33.33 |  |  | 3.03 |  |  |  | 4.87 |  |
| *GA8KES402I0XO3-129* | KWL | 7D | 47.99 |  |  |  | 3.03 |  |  |  | 4.86 |
| *GA8KES401CW5Y2-264* | KV | 7D | 54.12 | 3.88 |  |  |  | 6.43 |  |  |  |
| *GDRF1KQ01AYOU2-187* | KSA | 7D | 59.50 |  | 4.03 |  | 4.04 |  | 6.72 |  | 6.70 |
|  | KV | 7D | 59.50 |  | 3.77 |  | 3.98 |  | 6.24 |  | 6.60 |
| *GDS7LZN01CR25H-235* | KL | 7D | 60.34 |  | 3.28 |  | 4.25 |  | 5.41 |  | 7.22 |
| *contig02300-511* | KL | 7D | 61.05 |  | 3.85 |  | 3.13 |  | 6.64 |  | 5.23 |
| *GBUVHFX01AT5J8-167* | KSA | 7D | 63.18 |  | 3.49 |  |  |  | 5.71 |  |  |
|  | KV | 7D | 63.18 |  | 3.16 |  |  |  | 5.10 |  |  |
| *GBF1XID02IP0NJ-181* | KSA | 7D | 64.33 |  | 3.74 | 3.53 | 4.64 |  | 6.18 | 5.81 | 7.80 |
|  | KW | 7D | 64.33 |  | 3.09 | 3.70 | 4.30 |  | 4.98 | 6.12 | 7.17 |
|  | KV | 7D | 64.33 |  | 3.08 | 3.45 | 4.03 |  | 4.96 | 5.65 | 6.69 |
|  | HKW | 7D | 64.33 |  |  |  | 3.11 |  |  |  | 5.00 |
| *GCE8AKX02GFUOC-406* | KWL | 7D | 67.90 |  |  | 3.26 |  |  |  | 5.50 |  |
|  | KW | 7D | 67.90 |  |  |  | 3.61 |  |  |  | 6.13 |
| *GBF1XID01A7KWE-230* | KWL | 7D | 82.43 |  |  | 4.56 | 4.11 |  |  | 7.76 | 6.88 |
|  | KW | 7D | 82.43 |  |  | 3.24 |  |  |  | 5.29 |  |
| *GDS7LZN01EQ5QX-243* | KL | 7D | 94.34 |  | 3.32 | 3.48 | 4.96 |  | 5.42 | 5.72 | 8.42 |
|  |  |  |  |  |  |  |  |  |  |  |  |
|  | KSA | 7D | 94.34 |  | 3.20 |  |  |  | 5.18 |  |  |
| *BE493868-ATwsnp4* | KL | 7D | 94.34 |  | 3.32 | 3.48 | 4.96 |  | 5.42 | 5.72 | 8.42 |
|  | KSA | 7D | 94.34 |  | 3.20 |  |  |  | 5.18 |  |  |
| *GDRF1KQ02H7HJS-109* | KL | 7D | 94.35 |  | 3.36 | 3.74 | 5.11 |  | 5.71 | 6.43 | 9.02 |
|  | KSA | 7D | 94.35 |  | 3.14 |  |  |  | 5.26 |  |  |
| *GA8KES401ANMPM-140* | KSA | 7D | 96.59 | 3.38 |  |  |  | 5.51 |  |  |  |
|  | KV | 7D | 96.59 | 3.79 |  |  |  | 6.27 |  |  |  |
| *GBQ4KXB02H9FHP-217* | KV | 7D | 101.42 | 3.24 |  |  |  | 5.26 |  |  |  |
| *GB5Y7FA02JEVRI-292* | KWL | 7D | 128.07 | 3.35 | 4.16 |  | 3.74 | 5.46 | 6.97 |  | 6.16 |
| *GDRF1KQ01CJ4KM-378* | KSA | 7D | 246.23 |  | 4.63 |  | 4.58 |  | 7.83 |  | 7.70 |
|  | KV | 7D | 246.23 |  | 3.84 |  | 3.90 |  | 6.37 |  | 6.44 |
|  | HKW | 7D | 246.23 |  | 3.61 |  | 3.01 |  | 5.93 |  | 4.82 |
|  | KL | 7D | 246.23 |  | 3.31 |  | 3.02 |  | 5.40 |  | 4.85 |
|  | KW | 7D | 246.23 |  | 3.28 |  | 3.35 |  | 5.34 |  | 5.43 |
| *contig28852-433* | KSA | 7D | 248.28 |  | 4.63 |  | 4.58 |  | 7.83 |  | 7.70 |
|  | KV | 7D | 248.28 |  | 3.84 |  | 3.90 |  | 6.37 |  | 6.44 |
|  | HKW | 7D | 248.28 |  | 3.61 |  | 3.01 |  | 5.93 |  | 4.82 |
|  | KL | 7D | 248.28 |  | 3.31 |  | 3.02 |  | 5.40 |  | 4.85 |
|  | KW | 7D | 248.28 |  | 3.28 |  | 3.35 |  | 5.34 |  | 5.43 |
| *contig33612-254* | KSA | 7D | 254.32 |  | 4.63 |  | 4.58 |  | 7.83 |  | 7.70 |
|  | KV | 7D | 254.32 |  | 3.84 |  | 3.90 |  | 6.37 |  | 6.44 |
|  | HKW | 7D | 254.32 |  | 3.61 |  | 3.01 |  | 5.93 |  | 4.82 |
|  | KL | 7D | 254.32 |  | 3.31 |  | 3.02 |  | 5.40 |  | 4.85 |
|  |  |  |  |  |  |  |  |  |  |  |  |
|  | KW | 7D | 254.32 |  | 3.28 |  | 3.35 |  | 5.34 |  | 5.43 |
| *GDS7LZN02IRHPY-217* | KSA | 7D | 269.31 |  | 4.82 |  | 4.33 |  | 8.18 |  | 7.24 |
|  | KL | 7D | 269.31 |  | 3.82 |  | 3.51 |  | 6.35 |  | 5.75 |
|  | KV | 7D | 269.31 |  | 3.79 |  | 3.43 |  | 6.27 |  | 5.58 |
|  | HKW | 7D | 269.31 |  | 3.46 |  |  |  | 5.66 |  |  |
|  | KW | 7D | 269.31 |  | 3.21 |  | 3.16 |  | 5.20 |  | 5.08 |
| *contig30802-202* | KW | 7D | 334.63 | 3.76 |  | 3.26 | 3.79 | 6.17 |  | 5.31 | 6.23 |
|  | KV | 7D | 334.63 |  |  | 3.69 | 3.55 |  |  | 6.09 | 5.80 |
| *GDEEGVY02JBOY8-293* | KW | 7D | 410.66 | 3.62 |  |  |  | 5.92 |  |  |  |
|  | KV | 7D | 410.66 |  |  |  | 3.03 |  |  |  | 4.85 |
| *GA8KES401D6QO6-143* | KSA | 7D | 423.39 |  | 3.63 |  | 3.94 |  | 5.99 |  | 6.52 |
|  | KV | 7D | 423.39 |  | 3.21 |  | 3.54 |  | 5.20 |  | 5.79 |
|  | HKW | 7D | 423.39 |  | 3.17 |  |  |  | 5.13 |  |  |
|  | KW | 7D | 423.39 |  |  |  | 3.38 |  |  |  | 5.49 |
| *GBUVHFX01BEV0I-237* | KSA | 7D | 498.92 | 5.61 |  |  |  | 9.61 |  |  |  |
|  | KV | 7D | 498.92 | 9.89 |  |  |  | 17.14 |  |  |  |
| *GBB4FNX02F1UIU-187* | KL | 7D | 515.77 |  |  | 3.08 |  |  |  | 4.98 |  |
| *GDEEGVY01CX4EA-86* | KL | 7D | 515.78 |  |  | 3.08 |  |  |  | 4.98 |  |
| *BF483648-ATwsnp2* | KSA | 7D | 531.84 | 5.61 |  |  |  | 9.61 |  |  |  |
|  | KV | 7D | 531.84 | 9.89 |  |  |  | 17.14 |  |  |  |
| *GBF1XID02FYSIN-173* | KSA | 7D | 531.84 | 5.58 |  |  |  | 9.61 |  |  |  |
|  | KV | 7D | 531.84 | 9.83 |  |  |  | 17.12 |  |  |  |
| *GCE8AKX01CKH84-174* | KSA | 7D | 532.08 | 5.61 |  |  |  | 9.61 |  |  |  |
|  |  |  |  |  |  |  |  |  |  |  |  |
|  | KV | 7D | 532.08 | 9.89 |  |  |  | 17.14 |  |  |  |
| *GBUVHFX02G9LIA-70* | KSA | 7D | 532.10 | 5.61 |  |  |  | 9.61 |  |  |  |
|  | KV | 7D | 532.10 | 9.89 |  |  |  | 17.14 |  |  |  |
| *GBF1XID02I13RD-205* | KSA | 7D | 532.23 | 5.61 |  |  |  | 9.61 |  |  |  |
|  | KV | 7D | 532.23 | 9.89 |  |  |  | 17.14 |  |  |  |
| *F5XZDLF02JVJSS-244* | KSA | 7D | 532.79 | 5.61 |  |  |  | 9.61 |  |  |  |
|  | KV | 7D | 532.79 | 9.89 |  |  |  | 17.14 |  |  |  |
| *contig03102-479* | KSA | 7D | 533.76 | 5.61 |  |  |  | 9.61 |  |  |  |
|  | KV | 7D | 533.76 | 9.89 |  |  |  | 17.14 |  |  |  |
| *GBQ4KXB01A47IQ-31* | KV | 7D | 534.80 | 4.02 |  |  |  | 6.70 |  |  |  |
| *GA8KES402JKODN-129* | KV | 7D | 535.11 | 4.02 |  |  |  | 6.70 |  |  |  |
| *GDS7LZN02IRLO6-263* | KSA | 7D | 535.58 | 5.61 |  |  |  | 9.61 |  |  |  |
|  | KV | 7D | 535.58 | 9.89 |  |  |  | 17.14 |  |  |  |
| *contig37295-55* | KSA | 7D | 582.77 | 4.34 |  |  |  | 7.75 |  |  |  |
|  | KV | 7D | 582.77 | 4.80 |  |  |  | 8.72 |  |  |  |
| *GCE8AKX02ICIZ0-32* | HKW | 7D | 585.69 |  | 3.13 |  |  |  | 5.04 |  |  |

Abbreviation: Chr, chromosome, KL, kernel length; KW, kernel width; KV, kernel volume; KSA, kernel surface area; KWL, kernel width to length ratio; HKW, hundred-kernel weight; BLUP, best linear unbiased predictors ; PVE, phenotypic variation explained.
