# Supplementary material for: Genome-Wide Association Study of Kernel Traits in Aegilops tauschii
Source: Front Genet. 2021 May 28;12:651785. doi: 10.3389/fgene.2021.651785 (PMC8194309; doi:10.3389/fgene.2021.651785)
Supplement: Supplementary Table 7 — Genes Homologous annotation using KOBAS 3.0. [file Table_7.docx]

**Supplementary Table S7** Genes Homologous annotation using KOBAS 3.0.

| *A.tauschii* gene | Ricegene | Arabidopsis gene | Functional annotation |
| --- | --- | --- | --- |
| *AET7Gv20644900* | *Os08g0535800* | *NAC047* | *NAC* domain containing protein 47, *NAC* family proteins *NARS1/NAC2* and *NARS2/NAM* in the outer integument regulate embryogenesis in arabidopsis. |
| *AET2Gv20774800* | *Os12g0608600* | *MST1* | Mercaptopyruvate sulfurtransferase 1, sulfurtransferases 1 and 2 play essential roles in embryo and seed development in arabidopsis thaliana. |
| *AET4Gv20799000* | *Os10g0466300* | *MAC3B* | Encodes *MAC3B*, a U-box protein with homology to the yeast and human E3 ubiquitin ligase Prp19. Associated with the *MOS4*-associated complex (*MAC*), involved in plant innate immunity. |
| *AET5Gv20005900* | *Os03g0701700* | *ETR1* | Similar to prokaryote sensory transduction proteins contains a histidine kinase and a response regulator domain homodimer. Membrane component binds ethylene mutations affect ethylene binding and metabolism of other plant hormones such as auxin, cytokinins, *ABA* and gibberellic acid ethylene receptor. Has histidine kinase activity, is regulated by *RTE1* mutations in *ETR1* block ethylene stimulation of flavonol synthesis. |
| *AET5Gv20084100* | *Os08g0373000* | *ZAR1* | *ZAR1* was expressed in different tissues and affected organ size, while *ZAR8* was mainly expressed in grain tissues. |
| *AET5Gv21111600* | *Os11g0462500* | *APK1A* | Protein kinase capable of phosphorylating tyrosine, serine, and threonine residues. |
| *AET7Gv20364100* | *Os06g0179700* | *DBP1* | *DBP1* is involved in plant-potyvirus interactions, loss-of-function of *DBP1* renders resistance to potyviruses. |
| *AET7Gv20655900* | *Os12g0628500* | *MAP2B* | Encodes a *MAP2* like methionine aminopeptidase. |
| *AET1Gv20528800* | *Os10g0548800* | *TOC120* | Component of *TOC* complex, plastid protein import machinery. |
|  |  |  |  |
| *AET2Gv20774500* | *Os02g0831700* | *PLA2-BETA* | Involved in pollen development and germination and tube growth. Also involved in stomatal opening in response to light. |
| *AET4Gv20678300* | *Os04g0345800* | *ARD3* | Encodes a protein predicted to belong to the acireductone dioxygenase family. |
| *AET4Gv20678400* | *HSFA2C* | *HSFA1E* | Member of heat stress transcription factor (*Hsf*) family. |
| *AET4Gv20799100* | *Os04g0176400* | *scpl6* | Serine carboxypeptidase-like 6. |
| *AET5Gv21015900* | *Os11g0649600* | *ccb203* | Cytochrome biogenesis or f203, protein coding. |
| *AET5Gv21021300* | *Os03g0797400* | *TSA1* | Catalyzes the conversion of indole-3-glycerolphosphate to indole, the penultimate reaction in the biosynthesis of tryptophan. Functions as a heterocomplex with tryptophan synthase beta subunit (*TSA2*), and *TSA1* drive differentiation of constitutive and Inducible ER body formation in brassicaceae. |
| *AET5Gv20005800* | *Os02g0143100* | *SPP2* | Sucrose-6F-phosphate phosphohydrolase 2. |
| *AET4Gv20857900* | *Os03g0107900* | *FRA8* | Protein has a domain that shares significant similarity with the pfam03016 domain. It is expressed specifically in developing vessels and fiber cells, and *FRA8* is targeted to Golgi. Mutants have irregular xylem formation, reduced cellulose levels and plants are smaller than normal siblings. |
| *AET4Gv20858000* | *Os11g0656500* | *CDC25* | Encodes a homolog of yeast cell cycle regulator *CDC25.* It hascapable of reducing the mitotic cell length of transformed fission yeast. Non-plant *CDC25* proteins have been shown to do this. Also described as having arsenate reductase activity involved in arsenate resistance. |
| *AET4Gv20858300* | *Os05g0518000* | *FT* | FT, together with LFY, promotes flowering and is antagonistic with its homologous gene, *TERMINAL FLOWER1* (*TFL1*). *FT* is expressed in leaves and is induced by long day treatment. Either the *FT mRNA* or protein is translocated to the shoot apex where it induces its own expression. |
|  |  |  |  |
| *AET3Gv20999400* | *Os02g0731900* | *KCS6* | Encodes *KCS6*, a member of the 3-ketoacyl-CoA synthase family involved in the biosynthesis of *VLCFA* (very long chain fatty acids). |
| *AET6Gv20057000* | *Os07g0212200* | *CSP41A* | Encodes a protein with ribonuclease activity that is involved in plastid *rRNA* maturation. |
| *AET7Gv20143000* | *Os04g0551800* | *TCP-1* | Encodes a putative cytoplasmic chaperonin that is similar to mouse Tcp-1 (t complex polypeptide 1). |
| *AET7Gv20144600* | *Os06g0112000* | *CRR22* | Pentatricopeptide Repeat Protein containing the *DYW* motif，Required for editing of multiple plastid transcripts, Endonuclease activity. |
| *AET7Gv20807400* | *Os08g0107400* | *GONST1* | Encodes a Golgi-localized GDP-mannose transporter. It can transport ADP-glucose in vitro. |
| *AET7Gv20645300* | *Os08g0536000* | *MAB1* | Transketolase family protein. |
| *AET7Gv20648100* | *Os08g0538300* | *CERK1* | LysM receptor-like kinase Essential in the perception and transduction of the chitin oligosaccharide elicitor. Involved in chitin-mediated plant innate immunity, located in the plasma membrane. |
| *AET3Gv20637200* | *Os01g0686800* | *ATARCA* | Encodes the Arabidopsis thaliana homolog of the tobacco WD-40 repeat ArcA gene. Knockout mutants show insensitivity to gibberellin in GA-induced seed germination. |
| *AET3Gv20637300* | *Os01g0686400* | *AR401* | Unknown function. |
| *AET6Gv20201500* | *Os02g0125100* | *IIL1* | Encodes a methylthioalkylmalate isomerase involved in glucosinolate biosynthesis. |
| *AET7Gv20449600* | *Os06g0214850* | *NDC1* | *NAD(P)H* dehydrogenase C1. |
| *AET5Gv20748100* | *Os09g0535300* | *XCT* | *XAP5* family protein involved in light regulation of the circadian clock and photomorphogenesis. Nuclear localized. |
|  |  |  |  |
| *AET4Gv20695300* | *Os03g0393900* | *PLP4* | Patatin-related phospholipase A. Expressed in the floral gynaecium and is induced by abscisic acid (ABA) or phosphate deficiency in roots. |
| *AET6Gv20574900* | *Os02g0608300* | *FRS6* | Unknown function. |
| *AET6Gv20575000* | *Os02g0608400* | *LETM1* | *AtLETM1*; leucine zipper-EF-hand-containing transmembrane protein 1. |
| *AET5Gv21111700* | *EXPA7* | *EXP3* | PIL5, a phytochrome-interacting bHLH protein, regulates gibberellin responsiveness by binding directly to the GAI and RGA promoters in Arabidopsis seeds. |
| *AET6Gv20733800* | *MADS57* | *AGL44* | *MADS* box gene, transcription factor. Broad expression in roots after flowering (RPKM 19.3), leaves after flowering (RPKM 18.4). |
| *AET2Gv20682900* | *ASR6* |  | Unknown function. |
| *AET6Gv20201400* | *WRKY75* |  | Unknown function. |
